# An Anisotropic and Stable‐Conductance Patch for Mechanical–Electrical Coupling With Infarcted Myocardium

**DOI:** 10.1002/EXP.20250021

**Published:** 2025-12-16

**Authors:** Yimeng Li, Yuchen Miao, Leqian Wei, Wenxin Li, Mengqi Shan, Qianqian Jiang, Fujun Wang, Lu Wang, Ze Zhang, Jizhou Song, Yang Zhu, Jifu Mao

**Affiliations:** ^1^ Shanghai Frontiers Science Center of Advanced Textiles College of Lextiles Donghua University Shanghai China; ^2^ Key Laboratory of Textile Science and Technology Ministry of Education College of Textiles Donghua University Shanghai China; ^3^ Key Laboratory of Textile Industry for Biomedical Textile Materials and Technology Donghua University Shanghai China; ^4^ School of Materials Science and Engineering Shanghai Institute of Technology Shanghai China; ^5^ MOE Key Laboratory of Macromolecular Synthesis and Functionalization Department of Polymer Science and Engineering Zhejiang University Hangzhou China; ^6^ Key Laboratory of Soft Machines and Smart Devices of Zhejiang Province State Key Laboratory of Brain‐Machine Intelligence Department of Engineering Mechanics Zhejiang University Hangzhou China; ^7^ Département de Chirurgie, Faculté de Médecine Université Laval Québec Canada; ^8^ Axe Médecine Régénératrice Centre de Recherche du CHU de Québec, Université Laval Québec Canada; ^9^ State Key Laboratory of Transvascular Implantation Devices Hangzhou China

**Keywords:** anisotropic, bionic hierarchical structure, cardiac repair, mechanical–electrical coupling, stretchable

## Abstract

Polymeric conductive patches have conventionally been employed to facilitate the repair of infarcted myocardium by enhancing myocardial electrical conduction and providing mechanical support. However, it remains a challenge to restore the electrical conduction and diastolic–systolic functions with stable and anisotropic mechanical and electrical cues in the dynamic physiological environment. Herein, inspired by the hierarchical myocardial fiber microscopic striated structure, we established a weaving‐based processing method to compound a striated polypyrrole conductive coating on the surface of highly oriented elastic fiber bundles. This unique design endows the patch with exceptional stretchability (elongation at break > 400%), stable conductance (Δ*R*/*R*_0 _= 0.04 within 20% strain), and excellent fatigue resistance (Δ*R*/*R*_0 _= 0.01 after 1,000,000 cycles). In addition, the precision process grounded on woven molding accomplished the tunable mechanical and electrical properties of the patch, which facilitates the achievement of long‐term, stable, and anisotropic mechanical–electrical coupling with the infarcted myocardium. The rat MI model experiments demonstrated that this anisotropic conductive patch can not only improve cardiac function and electrical activity over an extended period, but also effectively inhibit myocardial inflammation and fibrosis and promote angiogenesis. This study proposes a promising MI‐treatment patch and highlights the potential of woven technology in processing biomaterials composed of both rigid and elastic materials.

## Graphical abstract

Inspired by the hierarchical structures of native myocardium, an anisotropic and conductance‐stable myocardial patch is designed. The patch, which aligns with the native tissue's anisotropic characteristics is engineered to provide infarcted myocardium with directionally electrical conduction and anisotropic mechanical support, achieving mechanical–electrical coupling. The biomimetic surface gives it stretchability, stable conductance, and fatigue resistance even under the heartbeat. This reduces post‐MI deterioration, providing a promising strategy for reconstructing infarcted myocardium.

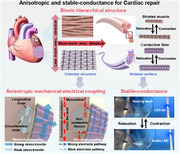

## 1 Introduction

Myocardial infarction (MI), a global health threat, ranks among the foremost contributors to morbidity and mortality, exacting a prodigious economic toll on society [1]. This pathological condition, precipitated by the occlusion of the coronary artery, sets off a complex cascade of events. Under normal physiological conditions, the myocardium exhibits highly coordinated electrical activity, characterized by synchronized action potentials initiated by the sinoatrial node and propagated through gap junctions (e.g., connexin‐43, CX‐43) [2]. This electrical synchronicity ensures efficient excitation‐contraction coupling, enabling rhythmic and forceful ventricular contractions to maintain systemic perfusion. However, upon ischemia‐induced apoptosis of electrically active cardiomyocytes (CMs), the erstwhile highly ordered myocardial tissue, due to the restricted regenerative potential of CMs, undergoes a progressive substitution by randomly deposited fibrotic tissue [3]. This pathological change results in typical disorders, including abnormal electrical communication and disparities in the distribution of cardiac pressure load caused by ventricular wall thinning, which gives rise to systolic and diastolic dysfunction, as well as arrhythmias [4]. Consequently, the restoration of the mechanical–electrical microenvironment within the post‐infarct myocardium has emerged as the primary objective in the pursuit of MI repair strategies.

To date, patches have been employed as mechanically supportive biomaterials with the aim of attenuating myocardial wall stress so as to passively impede ventricular enlargement [5]. The highly ordered structure of the natural myocardium enables it to exhibit anisotropic mechanical and electrical properties [6]. To further mimic this structure, alternative fabrication methods have been explored. Electrostatic spinning and 3D printing, for example, are employed to produce myocardial patches with highly oriented architectures, thereby emulating the biomimetic anisotropy of native myocardium and facilitating directional cell growth [7, 8]. In addition to mechanical support, endowing anisotropic myocardial patches with electrical activity holds the potential to re‐establish directional electrical coupling with the infarcted myocardium and ameliorate defective electrophysiology [9]. This is of paramount importance in preventing asynchronous ventricular contractions and malignant arrhythmias [10]. However, electroactive biomaterials, whether metallic, carbon‐based, or conductive polymers, are inherently rigid (> 1 GPa modulus) and brittle (< 10% elongation at break), which creates a mechanical mismatch between the elastic substrate and the flexible tissue [11, 12]. This results in the separation of the electroactive components from the composite patch under the contracting cardiac (> 20% strain). Consequently, the patch is incapable of maintaining stable electrical conductance during the contraction and relaxation of the heart and after the continuing cardiac cycles, which accordingly hinders the mechanical–electrical coupling with the host myocardium [13]. To compliantly respond to and mechanically interact with the cardiac deformation during both diastole and systole, patterned patches have been designed to further optimize the stretchable structure, thereby achieving elasticity and stable fatigue resistance [14, 15]. However, the stretchability of these patterned structures is achieved through the spatial deformation of the structures under strain, which is in conflict with the replication of the highly ordered architecture of myocardial tissue [16]. Consequently, there remains an unmet need for polymeric conductive patches that can simultaneously possess high stability and reasonable anisotropy under the dynamic physiological environment of cardiac systole and diastole [3]. Furthermore, although certain anisotropic electroactive patches have demonstrated favorable therapeutic effects in the context of MI, the precise nature of the mechanical–electrical interaction between these patches and the myocardium remains poorly understood [10]. For instance, it is crucial to elucidate the impact of the anisotropic mechanical properties of elastic patches on myocardial ventricular dilatation and the role of the stable electrical conduction properties of anisotropic patches in the restoration of synchronized electrophysiological propagation. Therefore, the development of cardiac patches capable of mechanical–electrical coupling with the infarcted myocardium persists as an urgent and pressing requirement.

In this study, we introduced an approach to fabricate a biomimetic hierarchical anisotropic conductive myocardial patch (HACMP) for post‐MI cardiac repair via woven molding. The unique hierarchical structure of HACMP, with oriented elastic fiber bundles and striated polypyrrole (PPy) coatings, mimicked the orientation and surface structures of the native myocardium. Such a biomimicking structure endowed HACMP with outstanding stretchability, stable conductivity, and fatigue resistance, enabling adaptation to the dynamic microenvironment of the heart. In vitro, HACMP showed tunable anisotropy for mechanical‐electrical coupling with myocardium, allowing directional electrical signal restoration and anisotropic mechanical support. In vivo rat MI model experiments confirmed HACMP's ability to restore electrical signals, inhibit cardiomyocyte apoptosis, provide mechanical support to prevent infarct expansion and fibrosis, promote angiogenesis, and modulate inflammation. These results suggest that HACMP represents a highly promising solution for MI treatment and highlight the potential and versatility of woven molding technology in customizing patches for clinical translation in myocardial repair.

## 2 Results and Discussion

### 2.1 Biomimetic Design of Anisotropic Patches

**FIGURE 1:**
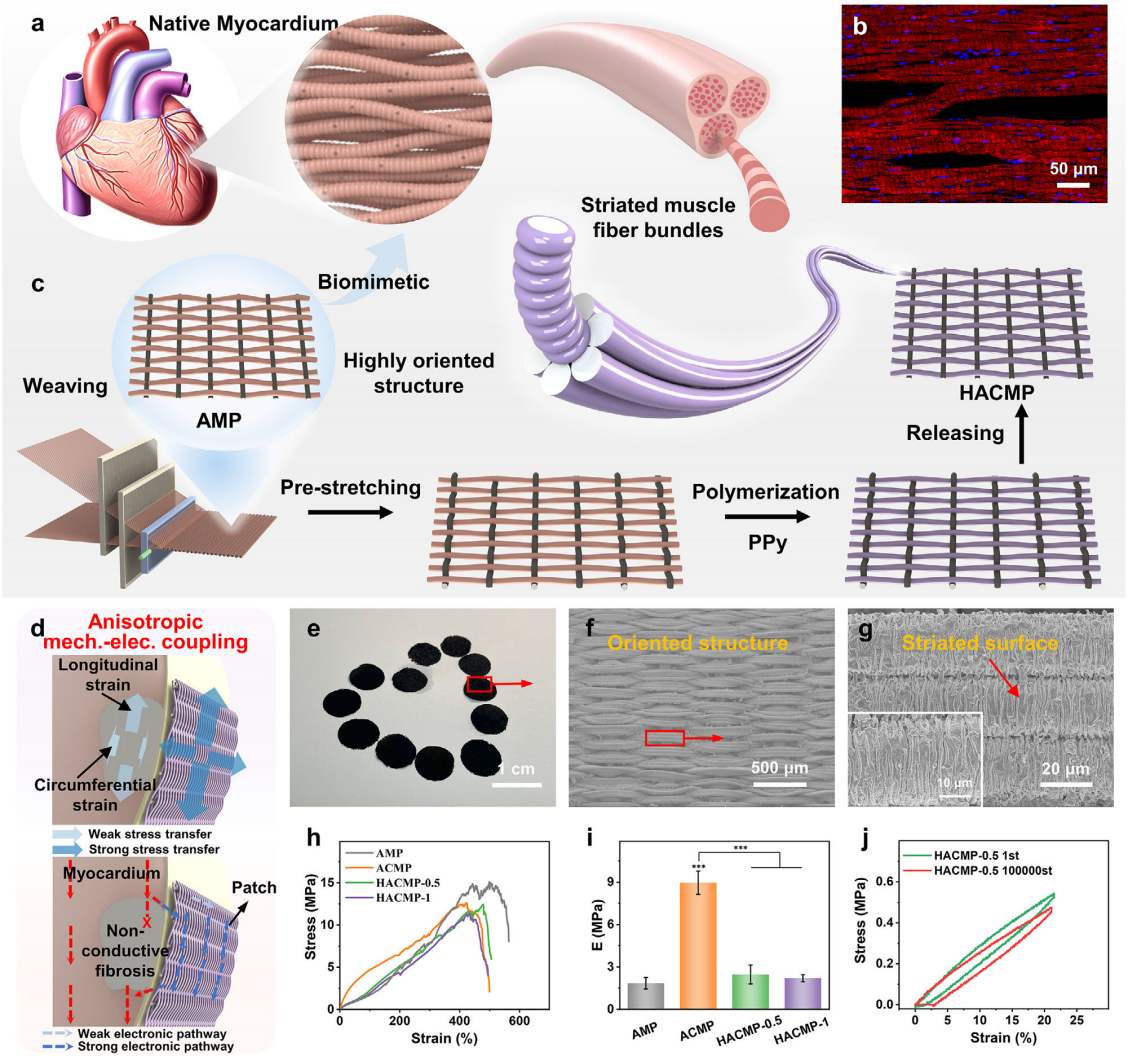
Biomimetic design of anisotropic patches for cardiac repair. (a) Schematic and (b) fluorescence micrographs of the multilevel structure of native cardiac tissue. (c) Schematic of HACMP production; (d) Schematic of mechanical–electrical coupling of HACMP to infarcted myocardium. (e) Optical picture and (f,g) SEM images of HACMP‐0.5. (h) Stress–strain curve and (i) Young's modulus of the patches. (j) Tensile cycling experiment of HACMP‐0.5 at 20% strain.

Natural myocardium is a multi‐level muscular tissue constituted by oriented striated muscle fiber bundles possessing elasticity and electrical activity (Figure 1a,b) [17]. It has a unique mechanical–electrical coupling microenvironment where the heart's contraction and relaxation, based on the movement between myosin and actin filaments in striated muscles as per Huxley's sliding filament theory, is closely associated with the action potential transmitted through gap junctions [18]. To replicate this microenvironment, a myocardial patch was designed following two principles. First, both elasticity and conductive stability are requisite to match the dynamic microenvironment of the myocardium, maintaining long‐term stability under 20% strain [19]. Second, it required an anisotropic structure and electromechanical property similar to the myocardium, with an anisotropy ratio from 1.9 to 3.9 [20].

To fulfill the first principle, polyurethane (PU) fiber bundles were chosen as the substrate for elasticity [21]. Among many conductive materials, PPy is widely used in the biomedical field due to its good biocompatibility and inherent conductivity [21, 22, 23]. Moreover, PPy has been shown to form strong interfacial interactions with PU substrates to avoid interfacial delamination [11]. Then, a striated PPy coating was fabricated on the substrate through a pre‐strain (*x* = 0, 0.5, 1) engineering, forming bioinspired striated muscle‐like electroactive fiber bundles (Figure 1c). The striated PPy coating demonstrated stable‐conductance in a dynamic physiological environment due to strain‐induced topological changes, similar to the surface changes of striated muscle during contraction and relaxation [16]. For the second principle, the weaving technique was employed to orient the bioinspired electroactive fibers, resulting in an oriented structure like the myocardial fiber bundles. Consequently, a bioinspired hierarchical structure‐featured anisotropic conductive myocardial patch (HACMP‐*x*) was developed. As depicted in Figure 1d, this patch enhances the mechanical compensation for the beating heart and improves the electrical coupling with the infarcted myocardium. Specifically, (1) the mechanical anisotropy backs the damaged site of the heart, aiding in the adaptation to heart deformation and restricting ventricular dilation; (2) the stable‐conductance and matching anisotropic conductivity accelerate electrical propagation in the MI area and supporting synchronous heart contraction by compensating for the impaired electrophysiology.

The HACMP, fabricated by industrial textile equipment, has good structural stability and can be tailored into any shape or size as per specific requirements. As shown in Figure 1e, the proposed patch was specifically cut into the common circular shape that has been prevalently adopted in the literature. SEM images of the patches reveal a clear structure of oriented fiber bundles along with a striated appearance on the fiber bundle surfaces, which closely resembles the hierarchical structure of the natural myocardial tissue (Figure 1f,g). As a control, a non‐conductive anisotropic myocardial patch without PPy coating (anisotropic myocardial patch, [AMP], Figure S1a) and an anisotropic PPy‐coated conductive myocardial patch without striated structure (ACMP, Figure S1b) were prepared. The weaving molding technique is essential for achieving the anisotropic oriented structure. In the woven patch, the weft direction was densely arranged, showing a distinct oriented structure, while the warp direction was sparsely arrayed and mainly used for assisting patch molding (Figure S1a). The striated PPy coating in the hierarchical structure was realized by pre‐strain engineering, and the larger the pre‐strain, the denser the striated structure (Figures S1c,d and S2), with a wavelength of 2.59 ± 0.85 µm at 50% pre‐strain. The characteristic peak at 993 cm^−1^ in the FTIR spectra corresponding to the in‐plane stretching vibration of ═C─H (Figure S3), indicating the successful polymerization of PPy [24].

Notably, the conductivity of conductive polymers relies on acid doping, which considerably impairs the patch's biocompatibility and conductivity stability in body fluid [10, 25]. As shown in Figure S4a, following a 14‐day incubation in PBS (0.1 M, pH 7.4), the conductivity of HACMP‐0.5 stabilized at 2.2 × 10^−4^ S cm^−1^, approximating that of cardiac tissue (10^−5^–10^−3^ S cm^−1^). XPS was employed to interpret the changes in the conductivity of the PPy coating before and after incubation. The chemical state of nitrogen (N1s) in the PPy main chain reveals the oxidation and protonation states of PPy. The N1s spectrum can be deconvoluted into four states: ═N─ at 398.0 eV, ─NH─ at 399.8 eV, ═N^+^─ at 401.4 eV, and ─NH^+^─ at 402.6 eV (Figure S4b,c) [12]. The relative content of the positively charged species decreased from 20.42% before incubation to 16.53% (Table S1). The changes in the oxidation and protonation states of PPy were mainly attributed to the doping of Cl^−^ and sulfosalicylic acid ions, as confirmed by the presence of S and Cl elements in the spectrum (Table S2). After incubation, the Cl element content decreased from 0.62% to 0.04%, while the S element content did not decline. This indicates that the decrease in the conductivity of the patch is mainly caused by the de‐doping of Cl^−^. It is confirmed that sulfosalicylic acid ions (as dopants) are hardly prone to precipitate out of PPy (Figure S4d), thus improving the stability of PPy in the physiological environment [13]. The patches utilized in this work were soaked in PBS for 2 weeks before use.

As illustrated in Figure 1h, owing to the aligned elastic PU core and the bioinspired striated PPy coating, all patches displayed remarkable stretchability in the weft direction (breaking elongation > 400%). Moreover, the weaving process and pre‐strain engineering did not affect the breaking strength and elongation at break of the patches (Figure S5). During stretching, the bioinspired striated PPy coating formed by the pre‐strain engineering unfolded, preventing the straightening of the rigid PPy coating, and resembling the straightening of initially wavy collagen fibers [15, 26]. Consequently, the Young's modulus of HACMP‐0.5 (2.47 ± 0.67 MPa) was much lower than that of the anisotropic conductive myocardial patch without striated surface (ACMP, 8.96 ± 0.83 MPa) (Figure 1i), and is comparable to the Young's modulus of the reported cardiac patches and cardiac tissue engineering scaffolds (Table S3). The cyclic and continuous beating of the myocardium demands high elasticity and fatigue resistance from myocardial patches. As shown in Figure 1j, during the loading and unloading process under 20% tensile strain, HACMP‐0.5 exhibited outstanding elasticity. Even after 100,000 cycles, the patch still retained 87% of its elastic deformation, which is significantly higher than the fatigue resistance of previously reported myocardial patches (Table S3).

### 2.2 Stabilized Conductance of HACMP in Dynamic Environments

**FIGURE 2:**
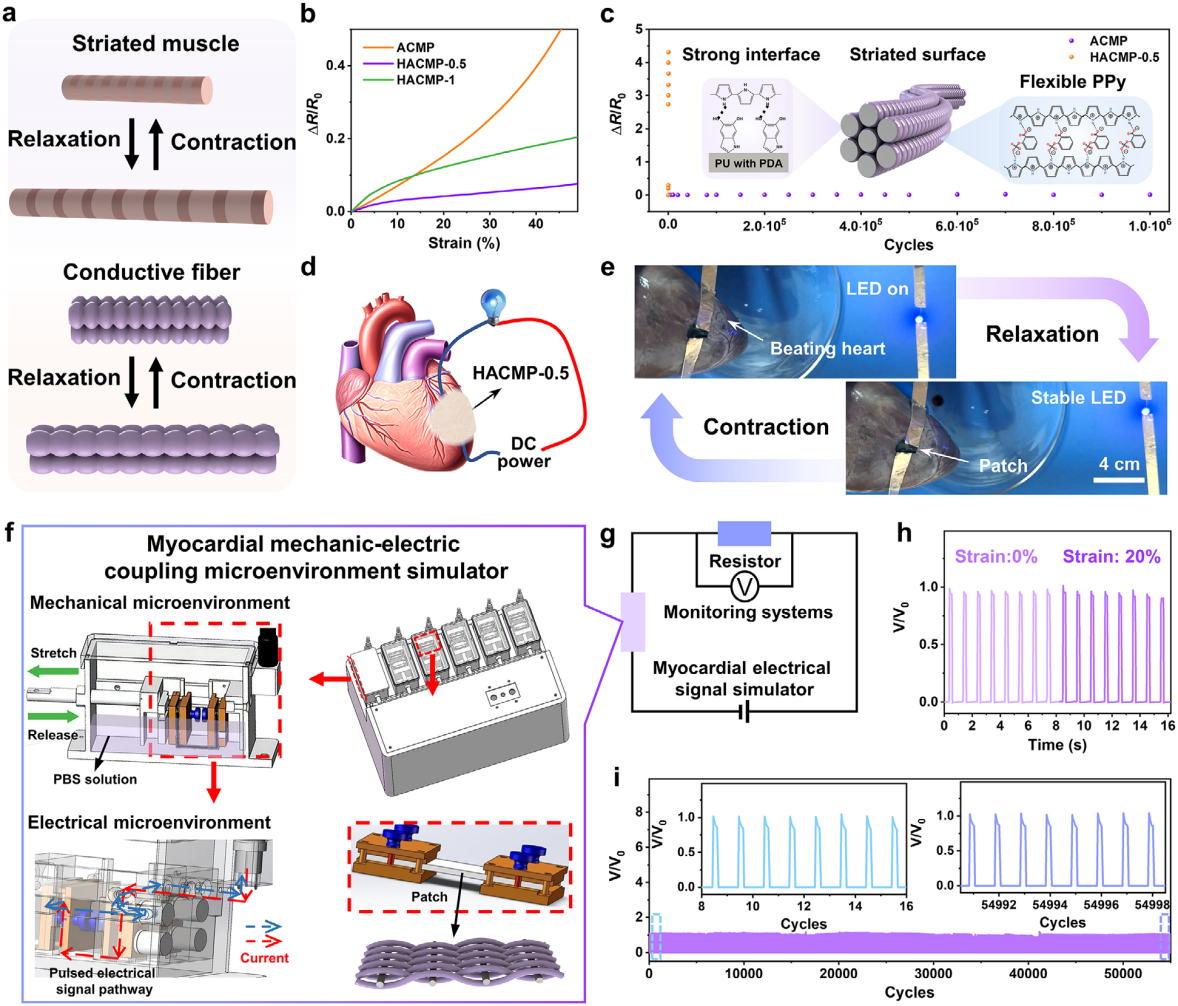
Stable conduction of hierarchical conductive myocardial patches in dynamic environments. (a) Schematic diagram illustrating the structural variations of the bionic striated PPy coating during cardiac systole and diastole. (b) Relative resistance changes under stretching of the conductive myocardial patch. (c) Cyclic endurance profile of the conductive myocardial patch at 20% strain. (d) Schematic illustration and (e) optical image of HACMP‐0.5, which is connected to a circuit containing an LED and glued on the surface of an isolated porcine heart. Schematic diagram of the (f) simulator and (g) system for the myocardial mechanical–electrical coupling microenvironment. (h) Performance of HACMP‐0.5 in conducting electrical signals with and without strain. (i) Performance of HACMP‐0.5 in stabilizing conductance electrical signals under 20% strain at 1 Hz.

Owing to the bioinspired stretchable structure on the surface of the hierarchical myocardial patch, HACMP‐0.5 exhibited the most excellent strain‐insensitive performance within 20% strain, with a Δ*R*/*R*_0_ of merely 0.04 (Figures 2a,b). In contrast, HACMP‐1, prepared under a higher pre‐strain, failed to exhibit enhanced strain‐insensitivity at 20% strain. The underlying cause could be the interfacial contact between the bioinspired PPy coating structures formed under high pre‐strain, as shown in Figure S1d. The initial electrical conductivity in the weft direction of HACMP‐1 (7.14 ± 0.70 × 10^−^⁴ S cm^−1^) was substantially greater than that of HACMP‐0.5 (2.97 ± 0.34 × 10^−^^−4^ S cm^−1^), further corroborating the contact between the striated structures. When the striated structures were stretched, as shown in Figure S6, the disappearance of contact resistance led to a sudden increase in resistance. This phenomenon, often exploited in sensors, was unfavorable for the stable transmission of electrical signals in the damaged myocardial area [27, 28]. The ACMP, lacking a striated surface structure, exhibited a relatively poor cyclic stability as its Δ*R*/*R*_0_ rose up to 4.31 after 600 cycles (Figure 2c). Conversely, owing to the combined effect of the inherent flexibility of the PPy coating, the stretchable biomimetic structural design, and the interfacial property enhancement induced by dopamine, as we previously reported [29], HACMP‐0.5 exhibited a minimal increment in Δ*R*/*R*_0_, merely 0.01, following 1 million stretching cycles. The bionic striated structure on the surface of HACMP‐0.5 did not peel off after 1 million tensile cycles (Figure S7), resulting in excellent strain‐insensitive performance over a 20% strain range (Figure S8). This performance remarkably surpasses that of previously reported myocardial patches (Table S4), thereby emphasizing its outstanding fatigue resistance. Furthermore, the two ends of the HACMP‐0.5 attached to the isolated heart were connected to a circuit equipped with an LED to verify its ability to conduct electrical signals during the cardiac cycle (Figure 2d). To mimic the heartbeat, a cyclic air supply was introduced into the heart chambers. As shown in Figure 2e and Video S1, HACMP‐0.5 was capable of upholding the brightness of the LED while the heart was beating, indicating stable electrical communication throughout the cardiac pulsation.

The synchronous contraction of the heart occurs through excitation‐contraction coupling after CMs receive and transmit electrical signals generated by the sinoatrial node [30]. Consequently, it is of paramount significance to simulate the mechanical–electrical coupling environment to assess the ability of patches to stably transmit electrical signals under physiological conditions. We have developed a simulator for the myocardial mechanical–electrical coupling microenvironment, with the specific details presented in Figure 2f and Figure S9. The simulator could independently apply electrical signals achieved by pulsed power and strain to the patch to simulate the myocardial microenvironment. A standard resistor (100 Ω) was connected in series throughout the circuit to prevent short‐circuiting and determine the change in the electrical signal on the patch by monitoring the voltage change across the standard resistor (Figure 2g). The electrical pulse signals monitored in the circuit were shown in the light‐colored square wave curve in Figure 2h without applying strain (0% strain). After applying the mechanical microenvironment (20% strain), the electrical signals monitored in the system remained almost unchanged, indicating that HACMP‐0.5 has the ability to maintain stable conduction of electrical signals in the mechanical–electrical coupling microenvironment. After 55,000 cycles of mechanical–electrical coupling stimulation under 20% strain and 1 Hz, the electrical signal conduction ability of HACMP‐0.5 remained almost unchanged (Figure 2i). This finding indicates that HACMP‐0.5 can stably restore the blocked electrical signals across the infarcted site within the dynamic environment of the beating heart. Thus, HACMP‐0.5 is the preferred choice for further investigations, unless otherwise stated.

### 2.3 Anisotropic Mechanical–Electrical Coupling of HACMP With Infarcted Myocardium

**FIGURE 3:**
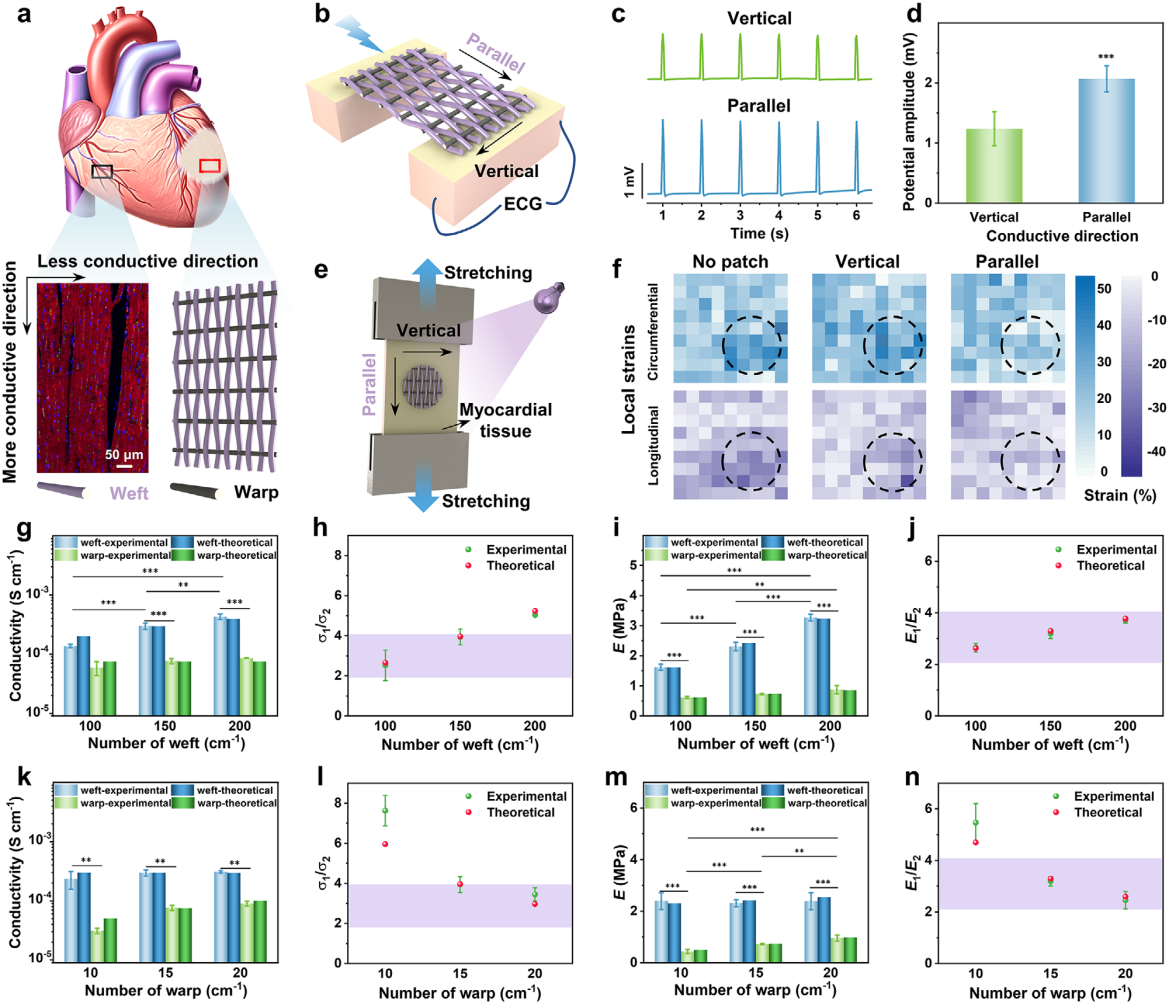
Mechanical–electrical coupling of tunable anisotropic conductive myocardial patches with the infarcted myocardium. (a) Schematic representation of the patch anisotropy aligned with the myocardium anisotropy. (b) Schematic diagram of the methodology for electromyographic testing of anisotropy patches. (c) Received electrical signals and (d) their associated intensity. (e) Schematic illustration of local strain measurement on infarcted myocardium pre‐ and post‐patch application. (f) Local strains in longitudinal and circumferential directions upon stretching the myocardium. Dashed circles: infarction. (g) Young's modulus and (h) anisotropy ratio of HACMP‐0.5 at a fixed warp density and variable weft densities. (i) Conductivity and (j) anisotropy ratio of HACMP‐0.5 at a fixed warp density and variable weft densities. (k) Young's modulus and (l) anisotropy ratio of HACMP‐0.5 with the same weft density and different warp densities. (m) Conductivity and (n) anisotropy ratio of HACMP‐0.5 with the same weft density and different warp densities. The purple band represents the range of the anisotropy ratio of the natural myocardial tissue. ***p < 0.001; **p < 0.01; and *p < 0.05.

Anisotropic patches with well‐defined properties can more precisely match the characteristics of native myocardial tissue [31]. This allows for mechanical integration with the host myocardium and effective transmission of electrical signals, as shown in Figure 3a. Consequently, a highly ordered patch is of great significance in cardiac treatment. In our study, the anisotropy of the patch was achieved by the precise arrangement of the striated PPy‐coated fiber bundles. The ability of HACMP‐0.5 to redirect electrical signals was assessed using electromyography (Figure 3b). When the orientation of the bionic fiber bundles in HACMP‐0.5 paralleled to that of the cardiac fiber bundles, the amplitude of the action potentials at recording sites was significantly higher than when the orientations were perpendicular (Figure 3c,d). These results suggest that in HACMP‐0.5, electrical signals mainly travel along the direction of the conductive fiber bundles, and the conductivity in the perpendicular direction is relatively low. In addition, applying anisotropic mechanical support to the infarcted myocardium can reduce pathological remodeling in the left ventricle and maintain cardiac function [14]. By tracking the movement of the fluorescent markers on the tissue, the effect of the anisotropic patch on the strain of the infarcted myocardium was quantitatively measured (Figure 3e), as we previously reported [32]. As depicted in Figure 3f, when a 20% strain was applied in the circumferential direction of the myocardium, the infarcted area was subjected to around 39.1% strain in the same direction. However, when HACMP‐0.5 was parallelly attached to the infarcted myocardium, the circumferential strain in the infarcted myocardium dropped significantly to 19.5%, which was much lower than the 33.4% when the orientation was perpendicular. This indicated that HACMP‐0.5 provides anisotropic mechanical support for the infarcted myocardium and better prevents cardiac stress concentration. In summary, HACMP‐0.5 was capable of achieving mechanical–electrical coupling with the anisotropic myocardium, thereby targeting restoration of electrical conduction and providing anisotropic mechanical support.

The optimal mechanical and electrical properties of cardiac patches remain a subject of contention, given the wide range of Young's modulus, conductivity, and their anisotropy in healthy natural heart tissues [33]. As a result, research on anisotropic myocardial patches with controllable mechanical–electrical properties for myocardial repair is valuable in formulating future strategies for treating MI. We employed an industrial weaving technology to fabricate anisotropic cardiac patches. This method permits easy adjustment of the reed gauge specification and winding speed, which respectively control the warp (*m*_1_) and weft densities (*m*_2_) of the patches, thereby granting patches diverse anisotropies (Figure S10). Theoretical prediction of the myocardial patch is a crucial precondition to attain tunable performance and aids in customizing patches to meet individualized requirements. Based on the geometrical deformation of the fiber‐based patch during stretching, Equation (1) (for more details on the derivation of the equation, see Supporting Information) was obtained to calculate the Young's modulus (*E*) and the anisotropy ratio of Young's modulus (*E*_1_/*E*_2_) for HACMP‐0.5.

(1) $$\left\{\begin{array}{c} E_{1}=0.250m_{1}+0.016m_{1}m_{2}\\ E_{2}=0.137m_{2}+0.016m_{1}m_{2} \end{array}\right.$$

The resistance of the HACMP‐0.5 mainly consists of two parts: the length resistance of the yarn and the contact resistance at the interlacing points. Assuming that the warp and weft yarns are interlaced perfectly perpendicular to each other, when ignoring the contact resistance at the interlacing points and only considering the length resistance of the warp and weft yarns, the resistive equivalent model of HACMP‐0.5 and its simplified unit were shown in Figures S11 and S12, respectively. Based on this model, a prediction model for the conductivity (*σ*) and the anisotropy ratio of conductivity (*σ*_1_/*σ*_2_) of HACMP‐0.5 was obtained as Equation (2) (for more details on the derivation of the equation, see Supporting Information).

(2) $$\left\{\begin{array}{c} \sigma_{1}=\frac{m_{1}}{20000+0.02m_{1}^{2}m_{2}}\\ \sigma_{2}=\frac{m_{2}}{500000+0.02m_{1}m_{2}^{2}} \end{array}\right.$$

The tensile and conductivity tests demonstrated that varying the warp and weft densities of the patch would adjust its Young's modulus, conductivity, and anisotropy ratio, as predicted by the theoretical model described above (Figures 3g–n). Specifically, the Young's modulus and conductivity of the myocardial patch in the weft direction increased significantly with increasing weft density. Moreover, altering the weft yarn density enabled the achievement of anisotropy in the myocardial patch, consistent with the anisotropy ratio of natural myocardial tissue [34]. Thus, the woven molding technique utilized in this study offers enhanced flexibility and control over the mechanical–electrical properties and their anisotropy, facilitating customization to suit the needs of patients of different ages or health conditions.

In conclusion, existing anisotropic myocardial patches, such as those fabricated via electrospinning, often achieve structural orientation but fail to replicate the hierarchical architecture of native myocardium, exhibiting poor fatigue resistance. Micropatterned patches constructed via 3D printing or electrowriting or hydrogel‐based patches prioritize stretchability at the expense of structural anisotropy (Tables S3 and S4). However, these approaches rarely achieve fatigue resistance beyond 1000 cycles, restricting their clinical utility in dynamic cardiac environments. In stark contrast, the oriented‐structured HACMP developed in this work demonstrates unparalleled durability, with a negligible resistance increase (Δ*R*/*R*_0_ = 0.01) after 1,000,000 cycles at 20% strain (Figure 2c). This exceptional performance stems from its hierarchical design: the striated PPy coating achieves strain dissipation through topological unfolding rather than rigid deformation. Furthermore, conventional fabrication methods like electrospinning and 3D printing face critical limitations in scalability and precision [3]. Electrospinning struggles with fiber alignment consistency, while 3D printing imposes trade‐offs between resolution and production speed. HACMP overcomes these challenges through industrial weaving technology, which aligns elastic PU fibers (Figure 1f). Consequently, this technology holds significant promise for mass production and distribution, potentially tailoring properties to individual patient needs for enhanced clinical application and efficacy.

### 2.4 Therapeutic Efficacy of the HACMP‐0.5 on MI

**FIGURE 4:**
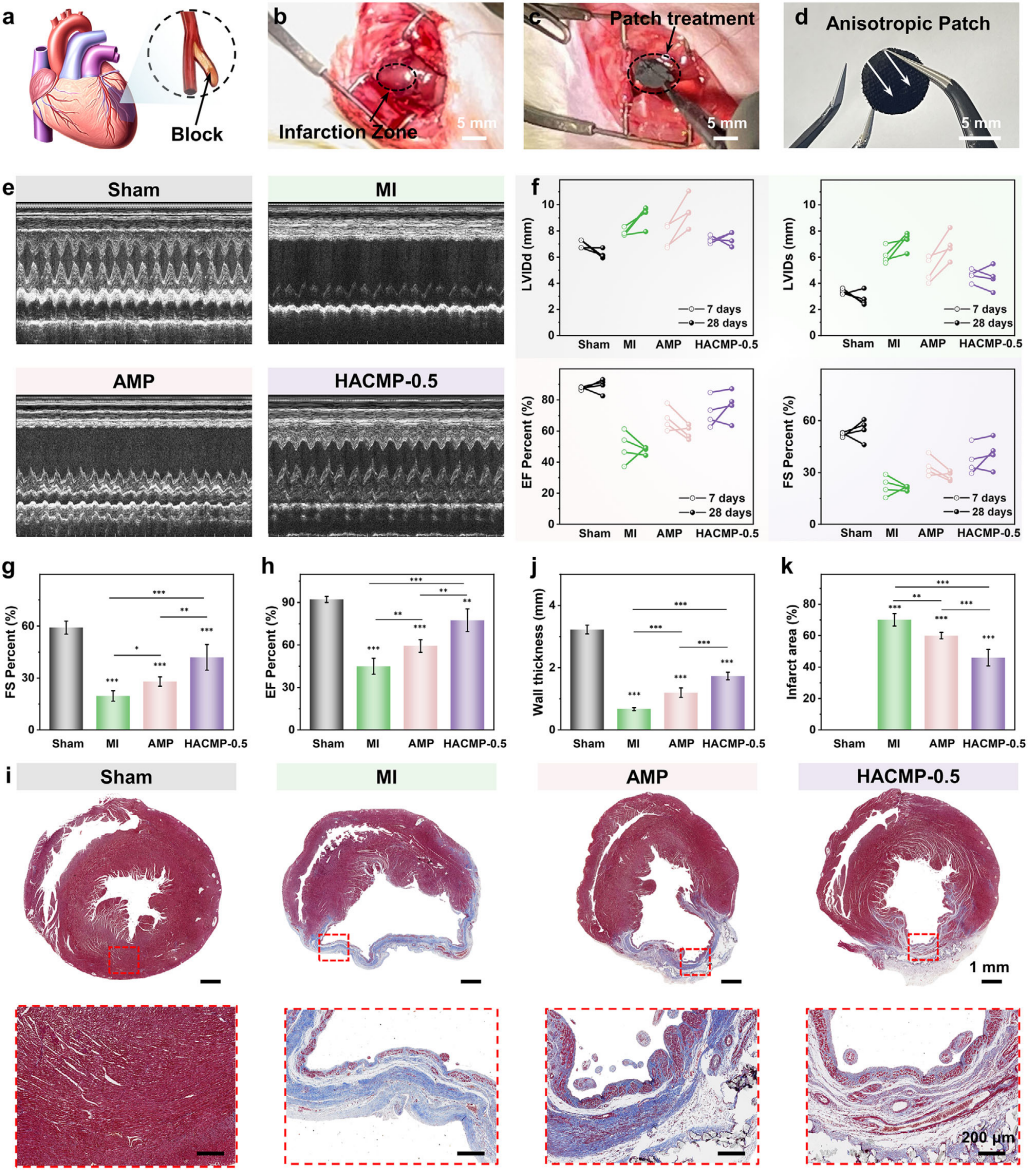
Therapeutic effect of anisotropic patch implantation on cardiac function recovery. (a) Schematic diagram of infarction resulting from myocardial ischemia. (b) Infarcted zone of the left ventricle following coronary artery ligation. (c) Image showing the myocardial patch implanted in the infarcted region. (d) Image of HACMP‐0.5 prior to implantation. (e) Echocardiography images of different groups. (f) Changes in LVIDd, LVIDs, FS, and EF in each group determined by echocardiography. Statistical analysis for (g) FS and (h) EF. (i) Masson's trichrome staining in different groups. Blue, fibrosis tissue; red, myocardium. Statistical analysis for (j) infarct wall thickness and (k) infarct area. *n* = 4; ***p < 0.001; **p < 0.01; and *p < 0.05).

An acute MI rat model was employed to assess the cardioprotective efficacy of the anisotropic myocardial patches (Figure 4a). Immediately subsequent to coronary artery ligation, the infarcted area became distinctly observable as a white region within the left ventricle. Figure S13 has been revised to present cell viability data (CCK‐8 assay), showing > 95% viability compared to controls, which quantitatively confirms the absence of cytotoxicity [35]. Upon verification of the outstanding biocompatibility of the anisotropic patches, the patches were surgically implanted in the infarcted heart (Figure 4b,c and Video S2) for myocardial repair performance evaluation. Figure 4d shows an image of HACMP‐0.5 prior to implantation.

Echocardiography was used to assess the therapeutic efficacy of the myocardial patch for repair of MI after 28 days of implantation (Figure 4e). In the MI group, postoperative left ventricular internal diameter at end‐diastole (LVIDd) and left ventricular internal diameter at end‐systole (LVIDs) were increased, while the ejection fraction (EF) and fractional shortening (FS) were lower compared to the sham‐operated group (Figure 4f), indicating cardiac impairment and left ventricular dilation. Figure 4e reveals that echocardiograms in the non‐conductive AMP and HACMP‐0.5 groups exhibited significant left ventricular systolic waves compared to the MI group, suggesting that anisotropic patch implantation improves cardiac function. Statistical analyses demonstrated that both AMP and HACMP‐0.5 were efficacious in preventing left ventricular infarct dilatation and deterioration of cardiac function compared with the MI group (Figure 4g,h). The HACMP‐0.5 group exhibited the best cardiac pumping function and prevention of adverse ventricular remodeling. After 28 days, compared with the MI group, the FS and EF of the HACMP‐0.5 group were enhanced by 95.45% and 60.19%, respectively. This indicated that HACMP‐0.5 could better achieve mechanical–electrical coupling with infarcted myocardium to maintain cardiac pumping function.

The therapeutic effect of the patch on the damaged myocardium was also investigated by Masson's trichrome staining. As shown in Figure 4i, the MI group presented malignant ventricular remodeling, evidenced by left ventricular dilatation, ventricular wall thinning, and massive deposition of collagen secreted by fibroblasts (blue) in lieu of myofibers composed of CMs (red). In comparison to the MI group, the AMP group showed an increase in ventricular wall thickness from 0.67 ± 0.05 to 1.20 ± 0.15 mm, and a reduction in infarct area from 70.08 ± 3.98% to 59.98 ± 2.07% (Figure 4j,k). The HACMP‐0.5 group demonstrated the most favorable repair outcomes, with an infarct area of 45.97 ± 5.22% and a wall thickness was 1.73 ± 0.12 mm, attesting to its superior capacity to alleviate fibrosis and inhibit ventricular dilation.

**FIGURE 5:**
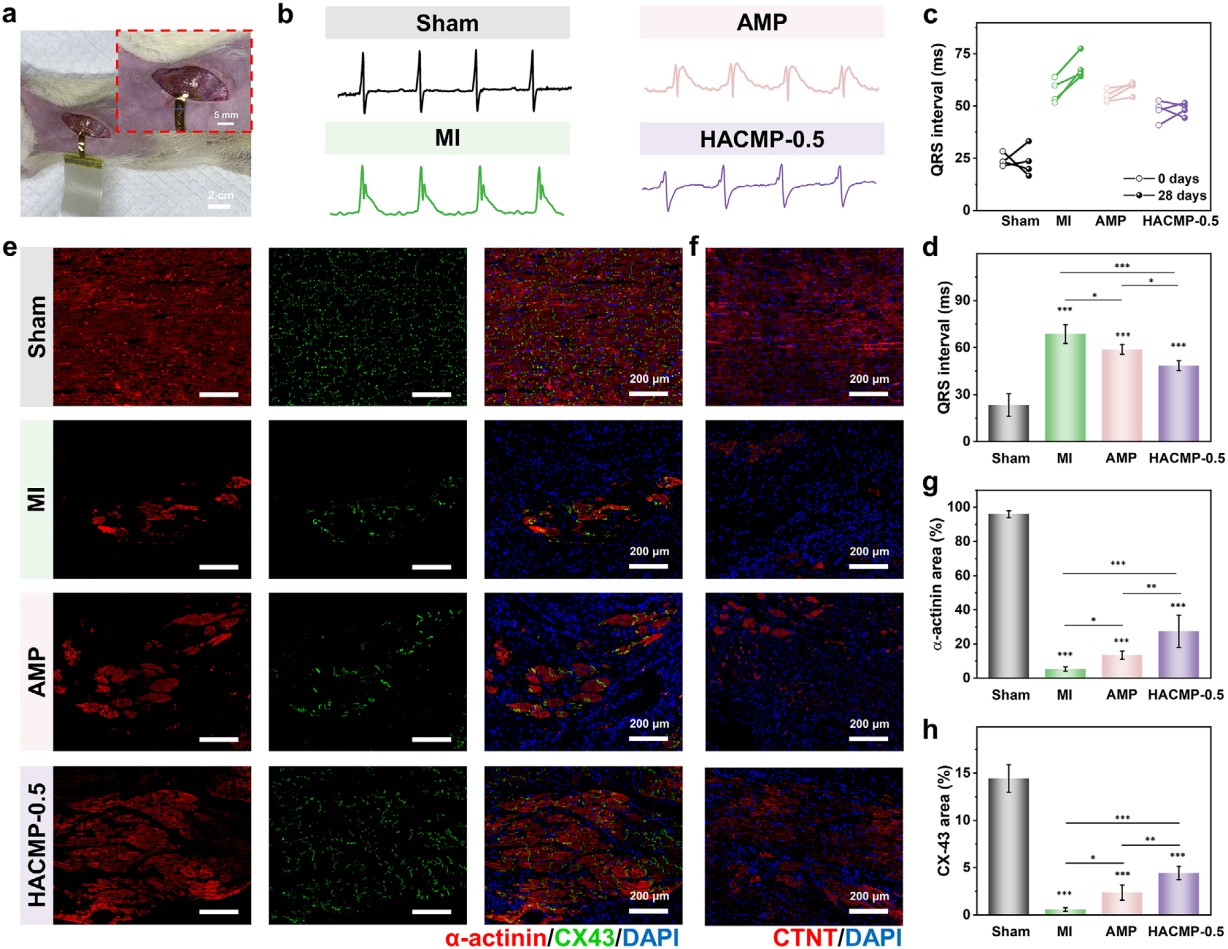
Electrocardiography (ECG) and electrophysiology after implantation of anisotropic patches. (a) Photographs of rats undergoing ECG tests. (b) ECG plots of different groups. (c) Changes in QRS interval as measured by ECG. (d) QRS interval duration. (e) Immunofluorescent staining of α‐actinin (red) and CX‐43 (green) proteins in the infarct area. (f) Immunofluorescent staining of cTnT (red) proteins in the infarct area. Percentage of (g) α‐actinin and (h) CX‐43 proteins calculated from immunofluorescent staining images. *n* = 4; ***p < 0.001; **p < 0.01; and *p < 0.05.

ECGs of different experimental groups were recorded to comprehensively characterize the cardiac electrophysiology subsequent to patch implantation (Figures 5a,b). Postoperatively, a significant prolongation of the QRS interval (the temporal duration from the Q wave to the S wave in the ECG) was observed in the MI group, signifying acute ischemic heart injury (Figure 5c). After a period of 28 days, as the disease progressed to the mature stage, notable ST‐segment elevation and further prolongation of the QRS interval were evident in the ECGs of both the MI and the AMP groups (Figure 5b). However, a markedly shorter QRS interval duration provided evidence that HACMP‐0.5 was capable of compensating for the electrical conductance spanning the infarcted area to the healthy regions (Figure 5d) [10].

To further evaluate the function of the surviving CMs in the infarcted myocardium, immunostaining for cardiac‐specific proteins was carried out. α‐actinin and cardiac troponin T (cTnT) are important cardiac proteins closely related to synchronized contraction of the myocardium [36]. Immunofluorescence staining revealed an increase in α‐actinin coverage from 5.34 ± 1.21% in the MI group to 13.46 ± 2.47% in the AMP group (Figure 5e,g), suggesting that the anisotropic patch inhibits myocardial fibrosis and cardiac remodeling by compensating for the mechanical disparities in infarcted myocardium. Notably, the expression of α‐actinin was maximized in the HACMP‐0.5 group, attaining a level of 27.44 ± 9.40%. Immunostaining for cTnT yielded analogous outcomes (Figure 5f). CX‐43, a gap junction protein in CMs, assumes a pivotal role in the electrical contraction coupling within the myocardium [37]. As shown in Figure 5e,h, the HACMP‐0.5 group manifested a higher expression level of CX‐43 protein (4.44 ± 0.70%) compared with the MI group and non‐conducting AMP group. This disparity reflects that the anisotropic conductive patch is more proficient in conducting synchronized communication and contraction of myocardial electrical signals [38]. Consequently, HACMP created an anisotropic, electrically coupled, and mechanically compensated dynamic microenvironment that enhanced intercellular electrical communication to inhibit fibrosis and promote myocardial repair.

**FIGURE 6:**
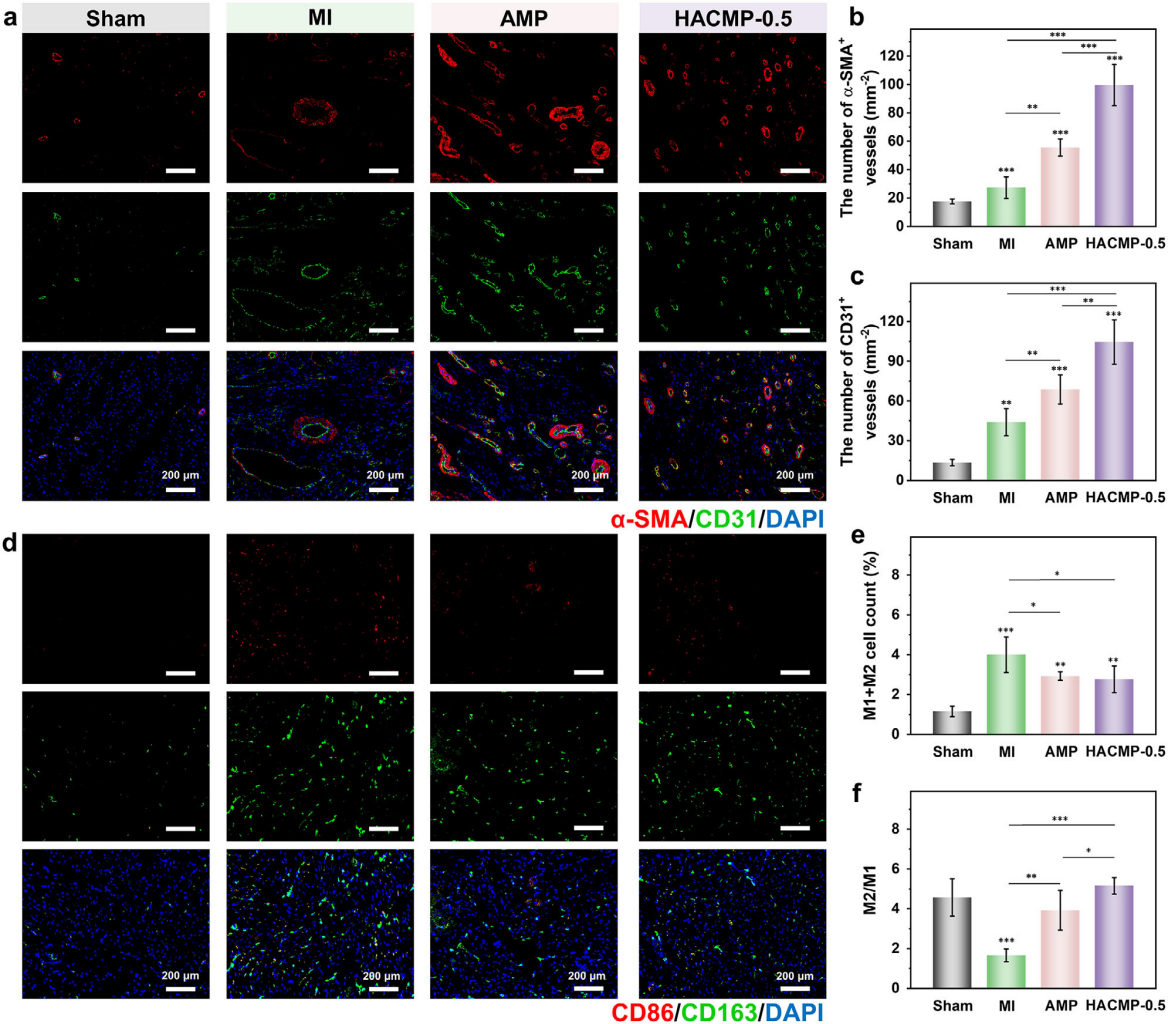
Anisotropic patches in host heart trigger low inflammation and boost revascularization. (a) Immunofluorescent staining of α‐SMA (red) and CD31 (green) proteins in the infarct area. Percentage of (b) α‐SMA and (c) CD31 proteins calculated from immunofluorescent staining images. (d) Immunofluorescent staining of CD86 (red) and CD163 (green) proteins in the infarct area. (e) Total M1 and M2 type macrophages and (f) M2/M1 type macrophage ratio calculated from immunofluorescent staining images. *n* = 4; ***p < 0.001; **p < 0.01; and *p < 0.05.

Blockage of the blood supply serves as the precipitating factor for MI [30]. Hence, revascularization is essential for restoring blood perfusion to residual CMs in the infarcted area and preventing further infarction progression. CD31, an endothelial cell‐specific marker, and α‐SMA, a vascular smooth muscle cell‐specific marker, were utilized to identify and quantify blood vessels in the infarcted myocardium [39]. In the MI group, large‐diameter small arterial vessels were detected in the infarcted myocardium (Figure 6a), a consequence of vascular compensation following capillary network disruption [40]. However, despite partial restoration of left ventricular blood flow, the hypoxic microenvironment of cardiomyocytes persisted due to capillary deficiency, resulting in significant cardiomyocyte loss and infarct area expansion [41]. Compared to the MI group, the AMP and HACMP‐0.5 groups exhibited notably elevated α‐SMA and CD31 expression levels (Figure 6b,c). The HACMP‐0.5 displayed the highest vascular density, potentially attributable to its anisotropic conductive network, which promotes cell adhesion and migration, facilitating the recruitment of endogenous endothelial cells [42].

Tissue regeneration is a complex process that creates a dynamic inflammatory milieu [36]. Post‐MI, necrotic myocardium releases abundant inflammatory factors, recruiting immune cells for inflammation resolution [43]. However, an intensified inflammatory reaction may trigger augmented cardiac remodeling and worsening cardiac function. Macrophages, crucial inflammatory cells, are pivotal in tissue repair and are classified into pro‐inflammatory M1 (high expression of CD86) and pro‐repair M2 types (high expression of CD163), with phenotypes reflecting tissue inflammation [44, 45]. In the MI group's infarcted regions, strong CD86‐positive (red) and CD163‐positive (green) fluorescent signals were noted (Figure 6d). Relative to the MI group, the total macrophage content in the AMP and HACMP‐0.5 groups declined by 26.75% and 32.50%, respectively (Figure 6e). Moreover, the ratio of M2/M1‐type macrophages was also conspicuously higher after implantation of anisotropic patches (Figure 6f), suggesting a propensity for macrophages to polarize into anti‐inflammatory M2 types. This polarization is conducive to impeding the deterioration of myocardial function during the late stage of infarction. The polarization of macrophages toward the M2 phenotype is mediated by the mechanical and biochemical microenvironment created by the patches: (1) The anisotropic structure of HACMP‐0.5 reduces pathological strain in the infarcted myocardium (circumferential strain decreased from 39.1% to 19.5%, Figure 3f), which mechanistically suppresses the activation of nuclear factor kappa‐light‐chain‐enhancer of activated B cells (NF‐κB). This transcription factor is a key driver of pro‐inflammatory cytokine production (e.g., TNF‐α, IL‐6). Concurrently, reduced strain promotes the activation of yes‐associated protein/transcriptional coactivator with PDZ‐binding motif (TAZ) pathways, known to induce anti‐inflammatory macrophage polarization [46, 47]. (2) The redox‐active polypyrrole backbone and the catechol groups of polydopamine endow the composites with efficient reactive oxygen species (ROS) scavenging ability. ROS are known to play a significant role in macrophage polarization, and their excessive production is often associated with a pro‐inflammatory state. By scavenging ROS, the PPy/polydopamine composites create a more favorable microenvironment for macrophage polarization towards the M2 phenotype [48].

In summary, HACMP‐0.5 facilitates myocardial repair through dual mechanisms. (1) Mechanical support—the orientation architecture mimics the native myocardium's mechanical anisotropy (anisotropy ratio: 1.9–3.9), reducing pathological strain in the infarcted region (Figure 3f). This attenuates ventricular wall stress and fibrotic remodeling (Figures 4i–k), as validated by reduced collagen deposition (infarct area: 45.97 ± 5.22% in HACMP‐0.5 vs. 70.08 ± 3.98% in MI group). (2) Electrical coupling—the stable conductance (Δ*R*/*R*_0_ = 0.04 within 20% strain) restores electrical propagation across the infarct, enhancing intercellular communication via CX‐43 gap junctions (Figures 5e–h). This synchronization shortens the QRS interval (Figure 5d), reducing arrhythmogenic triggers and improving contractile efficiency. These effects collectively inhibit fibrosis, promote angiogenesis, and modulate inflammation. Nevertheless, achieving an ideal repair of the infarcted myocardium remains challenging due to the limited regenerative capacity of cardiomyocytes [3]. Compared with the previously reported rigid and oriented tissue engineering scaffolds [49, 50, 51], the multilevel patch developed in this project can serve as a tissue engineering scaffold, offering an anisotropic environment that is more compatible with the mechanical–electrical microenvironment of the myocardium for seeded cells. The mechanical–electrical microenvironment simulator developed in this work also holds promise for application in a bioreactor, in combination with HACMP, to construct a tissue‐engineered myocardial patch with cardiac function.

## 3 Conclusion

In this study, a multifunctional anisotropic patch for infarcted myocardium repair was developed. The unique hierarchical structure of HACMP, which closely mimics the structure and performance of native myocardial tissue, endows it with outstanding mechanical and electrical properties. It exhibits remarkable stretchability, stable conductance even in dynamic environments, and excellent fatigue resistance. When it comes to mechanical–electrical coupling with the infarcted myocardium, the anisotropic characteristics of HACMP precisely align with those of the native tissue. This enables it to directionally transmit electrical signals and provide anisotropic mechanical support. In the acute MI rat model, HACMP‐0.5 demonstrated remarkable therapeutic efficacy. It not only improved cardiac function and reduced infarct size but also effectively inhibited fibrosis, promoted angiogenesis, and modulated the inflammatory response. In summary, HACMP holds substantial promise in the field of myocardial repair, representing a promising approach for MI treatment, and has the potential to significantly advance the development of cardiac repair technology.

## Author Contributions

**Yimeng Li**: conceptualization, methodology, formal analysis, writing – original draft. **Yuchen Miao**: methodology, formal analysis. **Leqian Wei**: investigation. **Wenxin Li**: visualization. **Mengqi Shan**: validation. **Qianqian Jiang**: formal analysis. **Fujun Wang**: resources. **Lu Wang**: supervision. **Ze Zhang**: supervision. **Jizhou Song**: resources. **Yang Zhu**: supervision, project administration, funding acquisition. **Jifu Mao**: writing – review and editing, supervision, project administration, funding acquisition. All authors have given approval to the final version of the manuscript.

## Conflicts of Interest

The authors declare no conflicts of interest.

## Ethics Statement

This project was approved by the experimental animal center of Donghua University (DHUEC‐STCSM‐2021‐14).

## Supporting information




**Supporting Information file 1**: exp270102‐sup‐0001‐SuppMat.docx.

**Supporting Information file 2**: exp270102‐sup‐0002‐VideoS1.mp4.

**Supporting Information file 3**: exp270102‐sup‐0003‐VideoS2.mp4.

## Data Availability

Data will be made available on request.

## References

[1] L. Zhang , T. Li , Y. Yu , et al., “An Injectable Conductive Hydrogel Restores Electrical Transmission at Myocardial Infarct Site to Preserve Cardiac Function and Enhance Repair,” Bioactive Materials 20 (2023): 339–354, 10.1016/j.bioactmat.2022.06.001. 35784639 PMC9210214

[2] J. Yang , L. Li , Y. Hu , Z. Li , and W. Hua , “Novel Electroactive Therapeutic Platforms for Cardiac Arrhythmia Management,” Advanced Science 12 (2025): 2500061, 10.1002/advs.202500061. 39951007 PMC12199593

[3] Y. Li , L. Wei , L. Lan , et al., “Conductive Biomaterials for Cardiac Repair: A Review,” Acta Biomaterialia 139 (2022): 157–178, 10.1016/j.actbio.2021.04.018. 33887448

[4] J. Zhan , X. Liao , X. Fan , et al., “An Injectable and Conductive Tempol/Polypyrrole Integrated Peptide Co‐Assembly Hydrogel Promotes Functional Maturation of Cardiomyocytes for Myocardial Infarction Repair,” Composites Part B‐Engineering 236 (2022): 109794, 10.1016/j.compositesb.2022.109794.

[5] S. Wang , Y. Yao , T. Zhou , et al., “Preservation of Cardiac Functions Post Myocardial Infarction In Vivo by a Phenylboric Acid‐Grafted Hyaluronic Hydrogel With Anti‐Oxidation and Accelerated Degradation Under Oxidative Microenvironment,” Composites Part B‐Engineering 238 (2022): 109941, 10.1016/j.compositesb.2022.109941.

[6] X. Jia , W. Liu , Y. Ai , et al., “A Multifunctional Anisotropic Patch Manufactured by Microfluidic Manipulation for the Repair of Infarcted Myocardium,” Advanced Materials 36, no. 44 (2024): 2404071, 10.1002/adma.202404071. 39279582

[7] G. Zhao , Y. Feng , L. Xue , et al., “Anisotropic Conductive Reduced Graphene Oxide/Silk Matrices Promote Post‐Infarction Myocardial Function by Restoring Electrical Integrity,” Acta Biomaterialia 139 (2022): 190–203, 10.1016/j.actbio.2021.03.073. 33836222

[8] Q. Lei , J. He , and D. Li , “Electrohydrodynamic 3D Printing of Layer‐Specifically Oriented, Multiscale Conductive Scaffolds for Cardiac Tissue Engineering,” Nanoscale 11, no. 32 (2019): 15195–15205, 10.1039/C9NR04989D. 31380883

[9] W. Xiong , X. Wang , H. Guan , et al., “A Vascularized Conductive Elastic Patch for the Repair of Infarcted Myocardium Through Functional Vascular Anastomoses and Electrical Integration,” Advanced Functional Materials 32, no. 19 (2022): 2111273, 10.1002/adfm.202111273.

[10] C. Yu , Z. Yue , M. Shi , et al., “An Intrapericardial Injectable Hydrogel Patch for Mechanical–Electrical Coupling With Infarcted Myocardium,” ACS Nano 16, no. 10 (2022): 16234–16248, 10.1021/acsnano.2c05168. 36190461

[11] Y. Li , Y. Gao , L. Lan , et al., “Ultrastretchable and Wearable Conductive Multifilament Enabled by Buckled Polypyrrole Structure in Parallel,” npj Flexible Electronics 6, no. 1 (2022): 42, 10.1038/s41528-022-00176-6.

[12] W. Li , Y. Li , M. Shan , et al., “Durable Flexible Conductive Fiber Based on Cross‐Linking Network Tannic Acid/Polypyrrole for Wearable Thermotherapy Monitoring System,” ACS Applied Materials & Interfaces 16, no. 36 (2024): 48329–48341, 10.1021/acsami.4c10302. 39189954

[13] Y. Li , C. Li , M. Shan , et al., “Injectable, Stretchable, and Conductance‐Stable Fiber for Myocardial Infarction Repair,” Composites Part B‐Engineering 273 (2024): 111242, 10.1016/j.compositesb.2024.111242.

[14] M. Castilho , A. van Mil , M. Maher , et al., “Melt Electrowriting Allows Tailored Microstructural and Mechanical Design of Scaffolds to Advance Functional Human Myocardial Tissue Formation,” Advanced Functional Materials 28, no. 40 (2018): 1803151, 10.1002/adfm.201803151.

[15] D. Olvera , M. S. Molina , G. Hendy , and M. G. Monaghan , “Electroconductive Melt Electrowritten Patches Matching the Mechanical Anisotropy of Human Myocardium,” Advanced Functional Materials 30, no. 44 (2020): 1909880, 10.1002/adfm.201909880.

[16] L. Wei , S. Wang , M. Shan , et al., “Conductive Fibers for Biomedical Applications,” Bioactive Materials 22 (2023): 343–364, 10.1016/j.bioactmat.2022.10.014. 36311045 PMC9588989

[17] J. M. Nerbonne and R. S. Kass , “Molecular Physiology of Cardiac Repolarization,” Physiological Reviews 85, no. 4 (2005): 1205–1253, 10.1152/physrev.00002.2005. 16183911

[18] G. Macchiarelli , O. Ohtani , S. A. Nottola , et al., “A Micro‐Anatomical Model of the Distribution of Myocardial Endomysial Collagen,” Histology and Histopathology 17, no. 3 (2002): 699–706. 12168777 10.14670/HH-17.699

[19] J. A. Cyr , M. Colzani , S. Bayraktar , et al., “Extracellular Macrostructure Anisotropy Improves Cardiac Tissue‐Like Construct Function and Phenotypic Cellular Maturation,” Biomaterials Advances 155 (2023): 213680, 10.1016/j.bioadv.2023.213680. 37944449

[20] L. M. Monteiro , F. Vasques‐Novoa , L. Ferreira , P. Pinto‐do‐O , and D. S. Nascimento , “Restoring Heart Function and Electrical Integrity: Closing the Circuit,” Npj Regenerative Medicine 2 (2017): 9, 10.1038/s41536-017-0015-2. 29302345 PMC5665620

[21] T. Chen , J. Cai , X. Cheng , S. Cui , D. Zhang , and D. Gong , “Bio‐Inspired Flexible Versatile Textiles for Excellent Absorption‐Dominated Electromagnetic Interference Shielding, Thermal Management, and Strain Sensing,” Chemical Engineering Journal 477 (2023): 147116, 10.1016/j.cej.2023.147116.

[22] X. Cheng , J. Cai , J. Xu , and D. Gong , “High‐Performance Strain Sensors Based on Au/Graphene Composite Films With Hierarchical Cracks for Wide Linear‐Range Motion Monitoring,” ACS Applied Materials & Interfaces 14, no. 34 (2022): 39230–39239, 10.1021/acsami.2c10226. 35988067

[23] Z. Sun , Q. Ou , C. Dong , et al., “Conducting Polymer Hydrogels Based on Supramolecular Strategies for Wearable Sensors,” Exploration 4, no. 5 (2024): 20220167, 10.1002/EXP.20220167. 39439497 PMC11491309

[24] Y. Li , M. Shan , J. Peng , et al., “A Highly Stretchable and Conductive Continuous Composite Filament With Buckled Polypyrrole Coating for Stretchy Electronic Textiles,” Applied Surface Science 610 (2023): 155515, 10.1016/j.apsusc.2022.155515.

[25] D. Mawad , C. Mansfield , A. Lauto , et al., “A Conducting Polymer With Enhanced Electronic Stability Applied in Cardiac Models,” Science Advances 2, no. 11 (2016): e1601007, 10.1126/sciadv.1601007. 28138526 PMC5262463

[26] Z. Dai , M. Lei , S. Ding , Q. Zhou , B. Ji , and M. Wang , “Durable Superhydrophobic Surface in Wearable Sensors: From Nature to Application,” Exploration 4, no. 2 (2023): 20230046, 10.1002/EXP.20230046. 38855620 PMC11022629

[27] Y. Wang , W. Qin , X. Hu , et al., “Hierarchically Buckled Ti_3_C_2_T* _x_ * Mxene/Carbon Nanotubes Strain Sensor With Improved Linearity, Sensitivity, and Strain Range for Soft Robotics and Epidermal Monitoring,” Sensors and Actuators B‐Chemical 368 (2022): 132228, 10.1016/j.snb.2022.132228.

[28] X. Cheng , J. Cai , P. Liu , T. Chen , B. Chen , and D. Gong , “Multifunctional Flexible MXene/AgNW Composite Thin Film With Ultrahigh Conductivity Enabled by a Sandwich‐Structured Assembly Strategy,” Small 20, no. 3 (2023): 2304327, 10.1002/smll.202304327. 37699748

[29] Y. Li , X. Liu , S. Wang , et al., “Dopamine‐Induced High Fiber Wetness for Improved Conductive Fiber Bundles With Striated Polypyrrole Coating Toward Wearable Healthcare Electronics,” Chemical Engineering Journal 485 (2024): 149888, 10.1016/j.cej.2024.149888.

[30] G. Tang , Z. Li , C. Ding , et al., “A Cigarette Filter‐Derived Biomimetic Cardiac Niche for Myocardial Infarction Repair,” Bioactive Materials 35 (2024): 362–381, 10.1016/j.bioactmat.2024.02.012. 38379697 PMC10876615

[31] C. Wang , Y. Chai , X. Wen , et al., “Stretchable and Anisotropic Conductive Composite Hydrogel as Therapeutic Cardiac Patches,” ACS Materials Letters 3, no. 8 (2021): 1238–1248, 10.1021/acsmaterialslett.1c00146.

[32] Y. Lu , T. Ren , H. Zhang , Q. Jin , L. Shen , and M. Shan , “A Honeybee Stinger‐Inspired Self‐Interlocking Microneedle Patch and Its Application in Myocardial Infarction Treatment,” Acta Biomaterialia 153 (2022): 386–398, 10.1016/j.actbio.2022.09.015. 36116725

[33] M. Kapnisi , C. Mansfield , C. Marijon , et al., “Auxetic Cardiac Patches With Tunable Mechanical and Conductive Properties Toward Treating Myocardial Infarction,” Advanced Functional Materials 28, no. 21 (2018): 1800618, 10.1002/adfm.201800618. 29875619 PMC5985945

[34] M. Montgomery , S. Ahadian , L. D. Huyer , M. Lo Rito , R. A. Civitarese , and R. D. Vanderlaan , “Flexible Shape‐Memory Scaffold for Minimally Invasive Delivery of Functional Tissues,” Nature Materials 16, no. 10 (2017): 1038–1046, 10.1038/nmat4956. 28805824

[35] Y. Zhu , X. Niu , T. Wu , et al., “Metal‐Phenolic Nanocatalyst Rewires Metabolic Vulnerability for Catalytically Amplified Ferroptosis,” Chemical Engineering Journal 485 (2024): 150126.

[36] L. Zhang , Z. Bei , T. Li , and Z. Qian , “An Injectable Conductive Hydrogel With Dual Responsive Release of Rosmarinic Acid Improves Cardiac Function and Promotes Repair After Myocardial Infarction,” Bioactive Materials 29 (2023): 132–150, 10.1016/j.bioactmat.2023.07.007. 37621769 PMC10444974

[37] K. Raniga , A. Nasir , N. T. N. Vo , et al., “Strengthening Cardiac Therapy Pipelines Using Human Pluripotent Stem Cell‐Derived Cardiomyocytes,” Cell Stem Cell 31, no. 3 (2024): 292–311, 10.1016/j.stem.2024.01.007. 38366587

[38] S. Zhu , C. Yu , N. Liu , et al., “Injectable Conductive Gelatin Methacrylate/Oxidized Dextran Hydrogel Encapsulating Umbilical Cord Mesenchymal Stem Cells for Myocardial Infarction Treatment,” Bioactive Materials 13 (2022): 119–134, 10.1016/j.bioactmat.2021.11.011. 35224296 PMC8844712

[39] E. Romano , I. Rosa , B. S. Fioretto , and M. Manetti , “The Contribution of Endothelial Cells to Tissue Fibrosis,” Current Opinion in Rheumatology 36, no. 1 (2024): 52–60, 10.1097/BOR.0000000000000963. 37582200 PMC10715704

[40] S. Apostolakis , G. Y. H. Lip , and E. Shantsila , “Monocytes in Heart Failure: Relationship to a Deteriorating Immune Overreaction or a Desperate Attempt for Tissue Repair?,” Cardiovascular Research 85, no. 4 (2010): 649–660, 10.1093/cvr/cvp327. 19805399

[41] Y. Shao , C. Xu , S. Zhu , et al., “One Endothelium‐Targeted Combined Nucleic Acid Delivery System for Myocardial Infarction Therapy,” ACS Nano 18, no. 11 (2024): 8107–8124, 10.1021/acsnano.3c11661. 38442075

[42] C. Song , X. Zhang , L. Wang , et al., “An Injectable Conductive Three‐Dimensional Elastic Network by Tangled Surgical‐Suture Spring for Heart Repair,” ACS Nano 13, no. 12 (2019): 14122–14137, 10.1021/acsnano.9b06761. 31774656

[43] Y. Que , J. Shi , Z. Zhang , et al., “Ion Cocktail Therapy for Myocardial Infarction by Synergistic Regulation of Both Structural and Electrical Remodeling,” Exploration 4, no. 3 (2023): 20230067, 10.1002/EXP.20230067. 38939858 PMC11189571

[44] S. Yan , M. Zhou , X. Zheng , et al., “Anti‐Inflammatory Effect of Curcumin on the Mouse Model of Myocardial Infarction Through Regulating Macrophage Polarization,” Mediators of Inflammation 2021 (2021): 9976912, 10.1155/2021/9976912. 34462629 PMC8403049

[45] W. Huang , Y. Tian , J. Ma , et al., “Neutrophil Membrane‐Based Biomimetic Metal‐Polyphenol Self‐Assembled Nanozyme for the Targeting Treatment of Early Brain Injury Following Subarachnoid Hemorrhage,” Chemical Engineering Journal 498 (2024): 155643, 10.1016/j.cej.2024.155643.

[46] Y. Zhu , Y. Matsumura , and W. R. Wagner , “Ventricular Wall Biomaterial Injection Therapy After Myocardial Infarction: Advances in Material Design, Mechanistic Insight and Early Clinical Experiences,” Biomaterials 129 (2017): 37–53, 10.1016/j.biomaterials.2017.02.032. 28324864 PMC5827941

[47] A. J. Engler , S. Sen , H. L. Sweeney , and D. E. Discher , “Matrix Elasticity Directs Stem Cell Lineage Specification,” Cell 126, no. 4 (2006): 677–689, 10.1016/j.cell.2006.06.044. 16923388

[48] T. Zhou , L. Yan , C. Xie , et al., “A Mussel‐Inspired Persistent ROS‐Scavenging, Electroactive, and Osteoinductive Scaffold Based on Electrochemical‐Driven In Situ Nanoassembly,” Small 15, no. 25 (2019): 1805440, 10.1002/smll.201805440. 31106983

[49] Y. Wu , L. Wang , B. Guo , and P. X. Ma , “Interwoven Aligned Conductive Nanofiber Yarn/Hydrogel Composite Scaffolds for Engineered 3D Cardiac Anisotropy,” ACS Nano 11, no. 6 (2017): 5646–5659, 10.1021/acsnano.7b01062. 28590127

[50] L. Wang , Y. Wu , T. Hu , B. Guo , and P. X. Ma , “Electrospun Conductive Nanofibrous Scaffolds for Engineering Cardiac Tissue and 3D Bioactuators,” Acta Biomaterialia 59 (2017): 68–81, 10.1016/j.actbio.2017.06.036. 28663141

[51] M. Alonzo , S. Anilkumar , B. Roman , N. Tasnim , and B. Joddar , “3D Bioprinting of Cardiac Tissue and Cardiac Stem Cell Therapy,” Translational Research 211 (2019): 64–83, 10.1016/j.trsl.2019.04.004. 31078513 PMC6702075

