# Supplementary material for: An Anisotropic and Stable‐Conductance Patch for Mechanical–Electrical Coupling With Infarcted Myocardium
Source: Exploration (Beijing). 2025 Dec 16;5(6):20250021. doi: 10.1002/EXP.20250021 (PMC12752566; doi:10.1002/EXP.20250021)
Supplement: Supplementary file 1 — Supporting Information file 1: exp270102‐sup‐0001‐SuppMat.docx. [file EXP2-5-20250021-s002.docx]

Supporting Information

**An anisotropic and stable-conductance patch for mechanical-electrical coupling with infarcted myocardium**

Yimeng Li^1,2,3,4^, Yuchen Miao^5^, Leqian Wei^1,2,3^, Wenxin Li^1,2,3^, Mengqi Shan^1,2,3^, Qianqian Jiang^6^, Fujun Wang^1,2,3^, Lu Wang^1,2,3^, Ze Zhang^7^, Jizhou Song^6^, Yang Zhu^5,8,*^, Jifu Mao^1,2,3,*^

^1.^ Shanghai Frontiers Science Center of Advanced Textiles, College of lextiles, Donghua University, Shanghai 201620, China.

^2.^ Key Laboratory of Textile Science & Technology, Ministry of Education, College of Textiles, Donghua University, Shanghai 201620, China.

^3.^ Key Laboratory of Textile Industry for Biomedical Textile Materials and Technology, Donghua University, Shanghai 201620, China.

^4.^ School of Materials Science and Engineering, Shanghai Institute of Technology, Shanghai 200235, China.

^5.^ MOE Key Laboratory of Macromolecular Synthesis and Functionalization, Department of Polymer Science and Engineering, Zhejiang University, Hangzhou 310027, China.

^6.^ Key Laboratory of Soft Machines and Smart Devices of Zhejiang Province, State Key Laboratory of Brain-Machine Intelligence, Department of Engineering Mechanics, Zhejiang University, Hangzhou 310027, China.

^7.^ Département de chirurgie, Faculté de médecine, Université Laval; Axe médecine régénératrice, Centre de recherche du CHU de Québec - Université Laval, Québec (QC), G1V 0A6, Canada.

^8.^ State Key Laboratory of Transvascular Implantation Devices, Hangzhou 310009, China.

^∗^ Jifu Mao

College of Textiles, Donghua University, 2999 North Renmin Road, Songjiang District, Shanghai 201620, China.

E-mail address: jifu.mao@dhu.edu.cn

Yang Zhu

MOE Key Laboratory of Macromolecular Synthesis and Functionalization, Department of Polymer Science and Engineering, Zhejiang University, Hangzhou 310027, China.

E-mail address: zhuyang@zju.edu.cn

**Supplementary Experimental Section**

**Materials:** Polyether polyurethane fiber bundles (40D/3f, 70D/6f) were obtained from Jiaxing Xinhai Textile Co., Ltd. (China). Pyrrole (Py, 99%, Aladdin) was purchased from Shanghai Yishi Chemical Co., Ltd. (China) and distilled twice under reduced pressure before use. Anhydrous ethanol (Analytical reagent), hydrochloric acid (HCl, Analytical reagent), sodium sulfosalicylate (NaSSA, Analytical reagent), iron trichloride hexahydrate (FeCl_3_·6H_2_O, Analytical reagent), tris(hydroxymethyl)methyl aminomethane (≥99%), and dopamine (DA, 98%) were purchased from Sinopharm Chemical Reagent Co., Ltd. (China).

**Fabrication of the HACMP.** The weaving technique was employed. Specifically, 70D PU fiber bundles were designated for the warp yarn, while 40D PU fiber bundles were opted for the weft yarn. By adjusting reed gauge specifications (controlling warp density, m₁) and winding speed (controlling weft density, m₂), anisotropic elastic fiber-based myocardial patches (AMP) were fabricated. The ends of the AMP in the weft direction with pre-stretching strain (*x*=0, 0.5, 1) were tied to a rectangular polymethyl methacrylate frame for fixation, followed by immersion in 2 mg mL^-1^ DA solution (0.05 M Tris-HCl buffer, pH=8.5) for 12 hours. Subsequently, the patch was rinsed and submerged in 20 mL aqueous solution of FeCl_3_·6H_2_O (0.36 M) and NaSSA (0.36 M) and then frozen at -20 °C. After that, 20 mL of cyclohexane solution containing Py (2.5 vol%) was layered on top of the frozen solution and allowed to undergo polymerization for 24 hours at 2 °C. The patch was then washed several times using anhydrous ethanol and deionized water. Finally, the pre-strain on the fiber was released to form the HACMP.

**Characterizations.** The morphologies of the patches were observed by scanning electron microscopy (SEM, Hitachi SU8010, Japan) operated at an accelerating voltage of 5 kV. The chemical composition of the patches was investigated by a Fourier transform near-infrared spectrometer at attenuated total reflectance mode (FTIR-ATR, Antaris II, Thermofisher, USA) and an X-ray photoelectron spectrometer (XPS, Escalab250xi, Thermofisher, USA) with Al Kα radiation (λ = 8.34 Å) as the excitation source. The mechanical properties of the fibers were tested using a universal material testing machine (LLY-06E, Laizhou Electron Instrument Co., Ltd., China). The gauge length was 5 mm and the stretching speed was 50 mm min^-1^. A source meter device (Keithley 2450, Tektronix, USA) was used to monitor the change in resistance of the conductive patches during deformation.

Place two myocardial tissues by a distance of 10 millimeters and position the sample between them. One of the myocardial tissues was connected to an electrical stimulator (with a voltage of 1 volt and a frequency of 1 hertz), while the other was linked to an electrophysiological signal recorder (Medlab-4C/501H, China) for recording electrical signals.

The myocardial tissue was unfolded along the circumferential direction and labeled with rhodamine B (with a distance of 1 mm from adjacent points in the x and y directions). The two ends of the myocardium with different patches attached were fixed onto the upper and lower fixtures of a universal mechanical testing machine (CTM2050, XieQiang Instrument Manufacturing Co., Ltd., China) and stretched to a strain of 20%. Photographs were taken of the relative displacements between the fluorescent spots under a UV lamp. The distances between adjacent points were measured according to the images corresponding to the samples before and after stretching. The local strains in the X and Y directions were calculated by comparing the corresponding distances and visualized using a color key.

**Cell viability assay.** Human foreskin fibroblasts (HFFs, Stem Cell Bank, Chinese Academy of Science, China) were seeded in a 24-well plate (2 × 10^4^ cells per well). The cell culture medium was formulated as 85% DMEM medium (Gibco, 11995065) and 15% fetal bovine serum (Gibco, 10099). The cells were then cultured in a direct heat carbon dioxide incubator (NU-5810E, NuAire Lab Equipment, USA) at a CO_2_ concentration of 5%, air concentration of 95%, and a temperature of 37 °C for 4 hours. Subsequently, patches, each measuring 8 mm in diameter, were placed into the wells. Following a co-incubation period of 72 hours, the viability of HFFs·was assessed using CCK-8 assay (Yeasen, China) following the manufacturer's protocol.

**Rat model of MI and implantation of HACMP.** The establishment of the rat model of myocardial infarction (MI) was carried out as follows. Briefly, adult male Sprague-Dawley rats (250 ±10 g) were anesthetized with 1% sodium pentobarbital (3 mg kg^-1^) by intraperitoneal injection and intubated for mechanical ventilation (tidal volume = 5 mL kg^-1^, frequency = 80 min^-1^). Thoracotomy was performed in the fourth intercostal space through the left parasternal incision to expose the heart. An excision was made at the pericardium to visualize the left anterior descending (LAD) branch of the coronary artery, which was then permanently ligated with a 6.0 suture at 2 - 3 mm between the left atrium and the pulmonary artery conus to create MI. Effective ligation was confirmed by observing the whitening of the heart’s anterior wall and apex. In the sham operation group, the procedure was done identically except that the suture was passed under the LAD coronary artery but not tied. The rats were randomly divided into the following groups: control (sham operation, n=4), MI (n=4), MI + AMP (n=4), and MI+HACMP-0.5 (n=4) groups. The rats were not medicated before or after surgery, and were fed a normal diet. Immediately after ligation, an 8-mm-diameter circle of AMP and HACMP-0.5 was sutured to the infarcted area of the anterior wall of the left ventricle. Finally, the incision in pericardium was sutured and chest was closed, and the rats were allowed to recover. This project was approved by the experimental animal center of Donghua University (DHUEC-STCSM-2021-14).

**Echocardiography.** The cardiac function of the rats was measured by echocardiography using a small-animal high-resolution ultrasound imaging system (Vevo2100, Visual Sonics, Toronto, Canada). During the echocardiogram, the rats were anesthetized with 1.5% isoflurane. The left ventricular ejection fraction (LVEF), fractional shortening (FS), left ventricle during end-diastolic (LVIDd) and end-systole (LVIDs) were measured in M-mode.

**Histology analysis.** Heart ventricular tissues were harvested and fixed with 4% paraformaldehyde for at least 24 hours before being processed for paraffin embedding. 4-μm thick sections were cut and stained with H&E and Masson's trichrome agents following the well-established protocols (n = 4, and 6 pictures from different areas were selected for each group).

**Immunofluorescence analysis****.** Immunofluorescence analysis was carried out as follows. Firstly, after antigen retrieval of the sections, the samples were incubated with a 3% bovine serum albumin (BSA, ST023, Beyotime Biotechnology Co., Ltd., China) in PBS solution at 37 °C for 30 minutes for blocking. Subsequently, primary antibodies were added dropwise and the samples were incubated overnight at 4 °C. The primary antibodies used were mouse monoclonal anti-cTnT antibody (Abcam, catalog number ab8295), mouse monoclonal anti-α-actinin antibody (Abcam, catalog number ab68194), rabbit monoclonal anti-CX-43 antibody (Proteintech, 26980-1-AP), rabbit monoclonal anti-CD31 antibody (Abcam, catalog number ab182981), mouse monoclonal anti-α-SMA antibody (Abcam, catalog number ab7817), mouse monoclonal anti-CD86 antibody (Abcam, catalog number ab220188) and rabbit monoclonal anti-CD163 antibody (Abcam, catalog number ab182422). After that, the samples were washed with PBS and then incubated with fluorescence-labeled secondary antibodies (Alexa Fluor®594, ab15011; Alexa Fluor®594, ab150080; Alexa Fluor®488, ab150113; Alexa Fluor®488, ab150077) for 30 minutes. Finally, the cell nuclei were stained with DAPI (Thermo Fisher, D1306) for 10 minutes. Photographs were taken using a confocal microscope. Image analysis was performed using Image J software to calculate the percentage of positive cells (n = 4, and 6 pictures from different areas were selected for each group).

**Statistical analysis.** The data were analyzed by means of one-way analysis of variance (ANOVA) accompanied by LSD post-test within the IBM SPSS Statistics 26 software. Error bars mean ± SD. The significant difference was marked when p < 0.001 (***), p < 0.01 (**), p < 0.05 (*).

**Supplementary Figures**


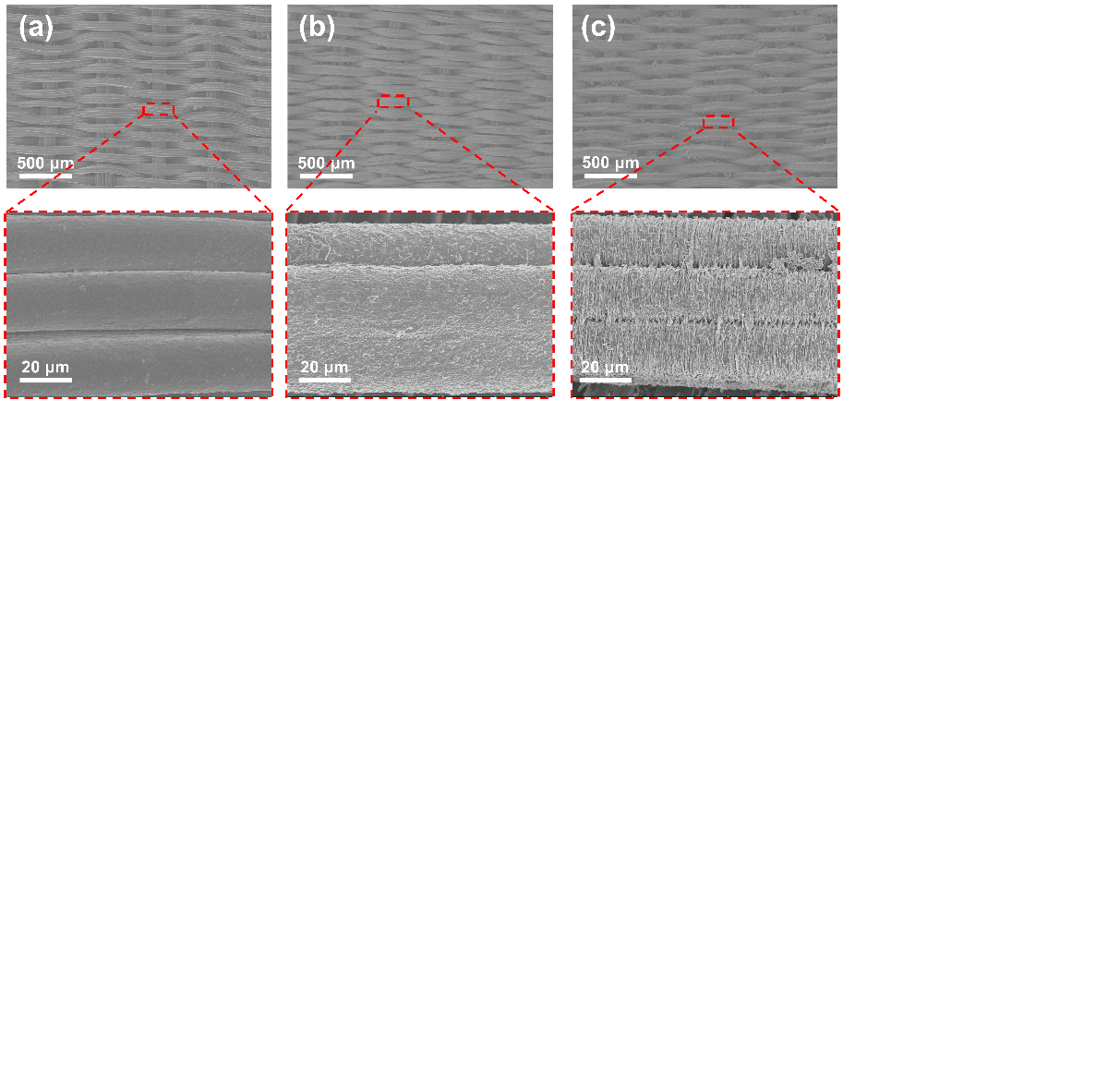


**Figure S1** SEM images of (a) AMP, (b) ACMP, and (c) HACMP-1.

**
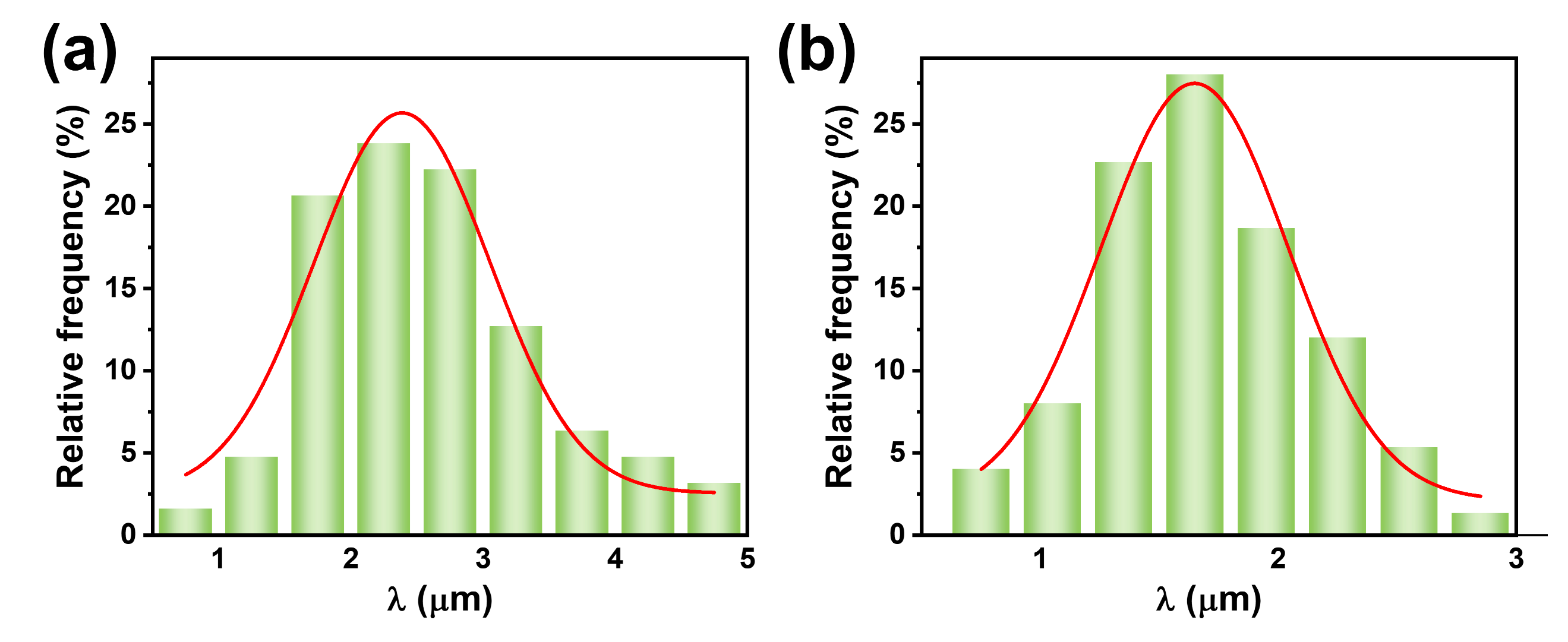
**

**Figure S2** Relative frequencies of the wavelengths of the striated structure on (a) HACMP-0.5 and (b) HACMP-1 measured from SEM images.

**
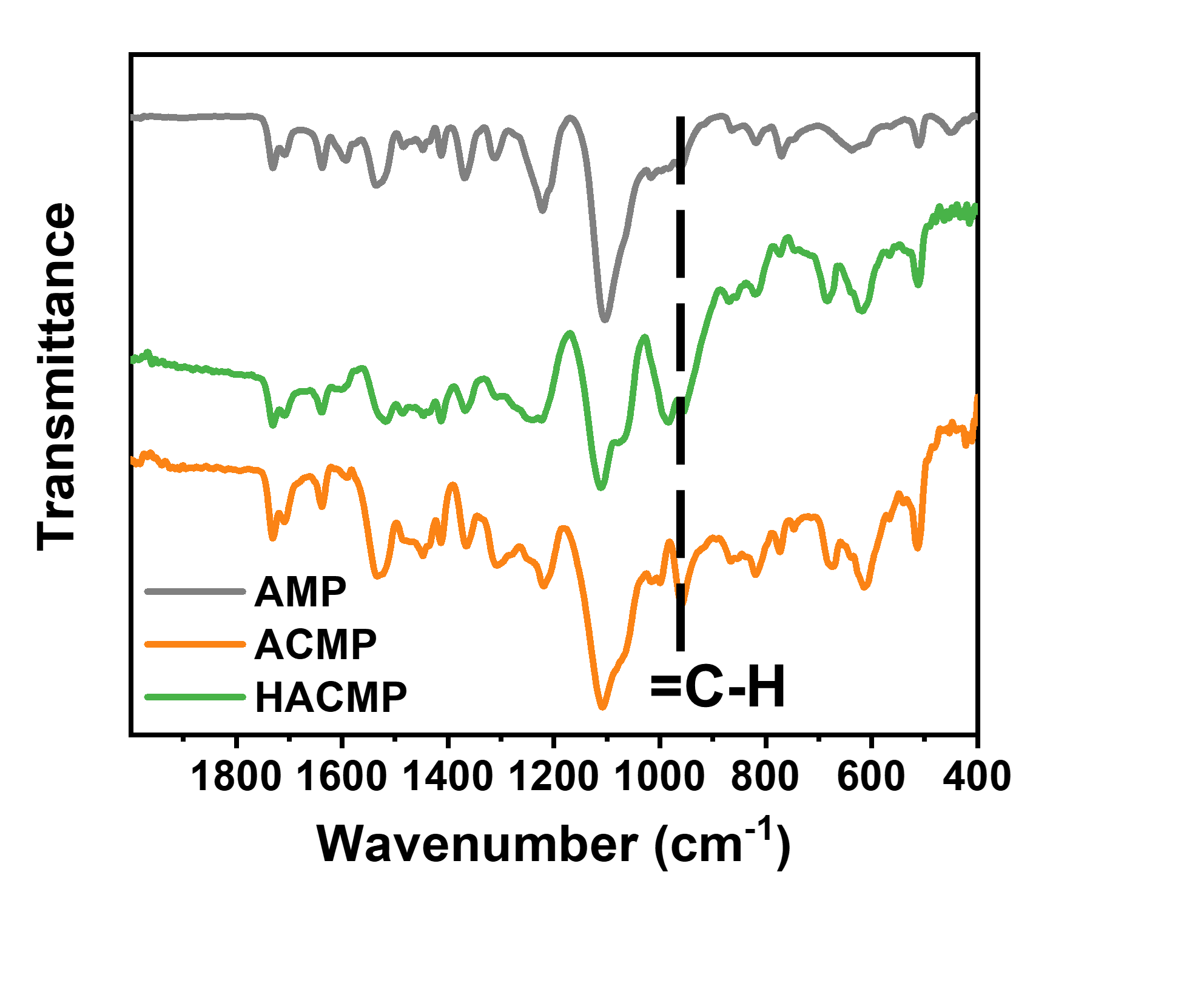
**

**Figure S3** FTIR spectra of patches.

**
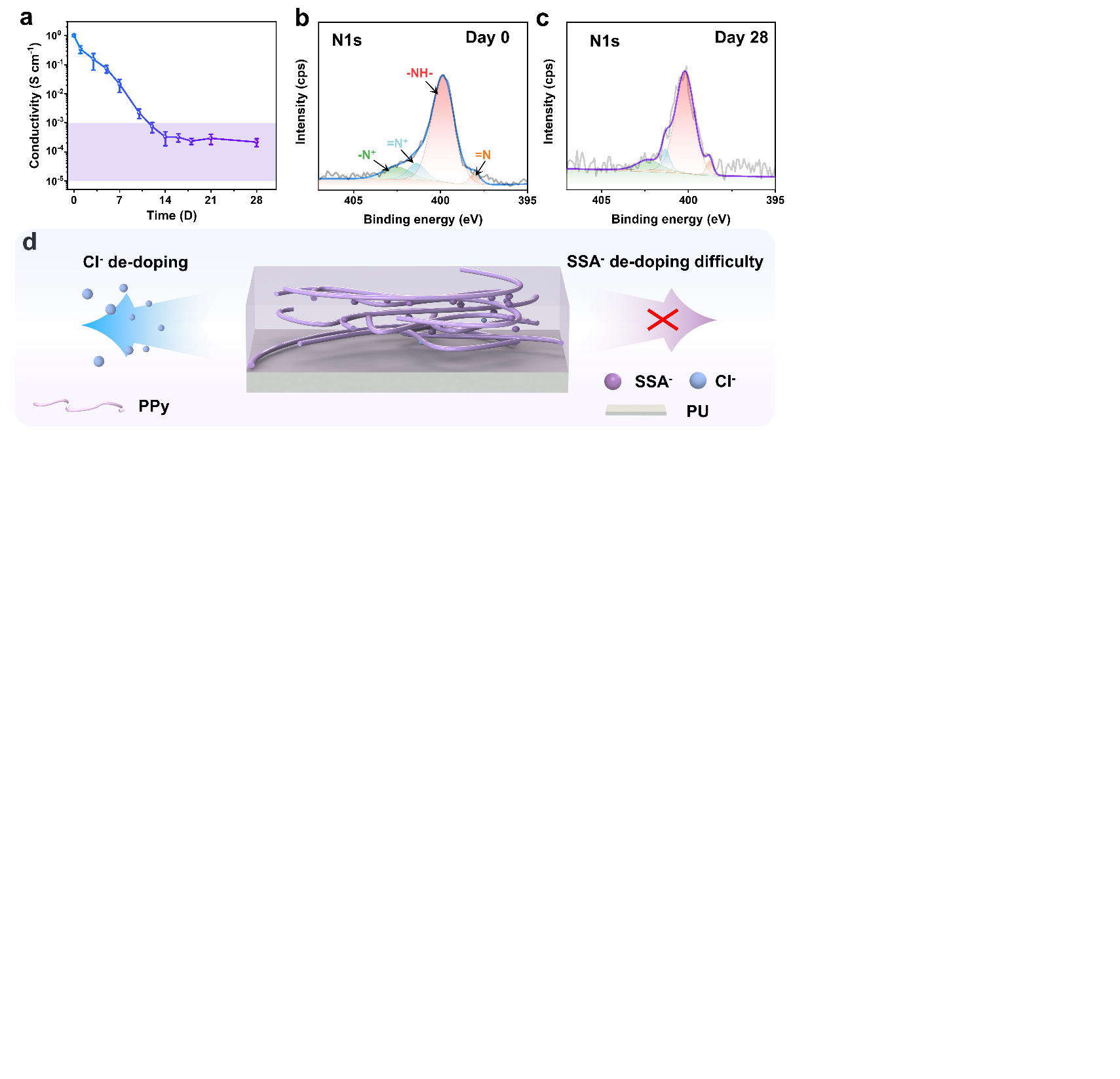
**

**Figure S4** (a) Conductivity changes of HACMP-0.5 in PBS. High-resolution XPS spectra of N1s of HACMP-0.5 (b) before and (c) after incubation. (d) Schematic representation of the de-doping of the PPy coating after incubation.

**
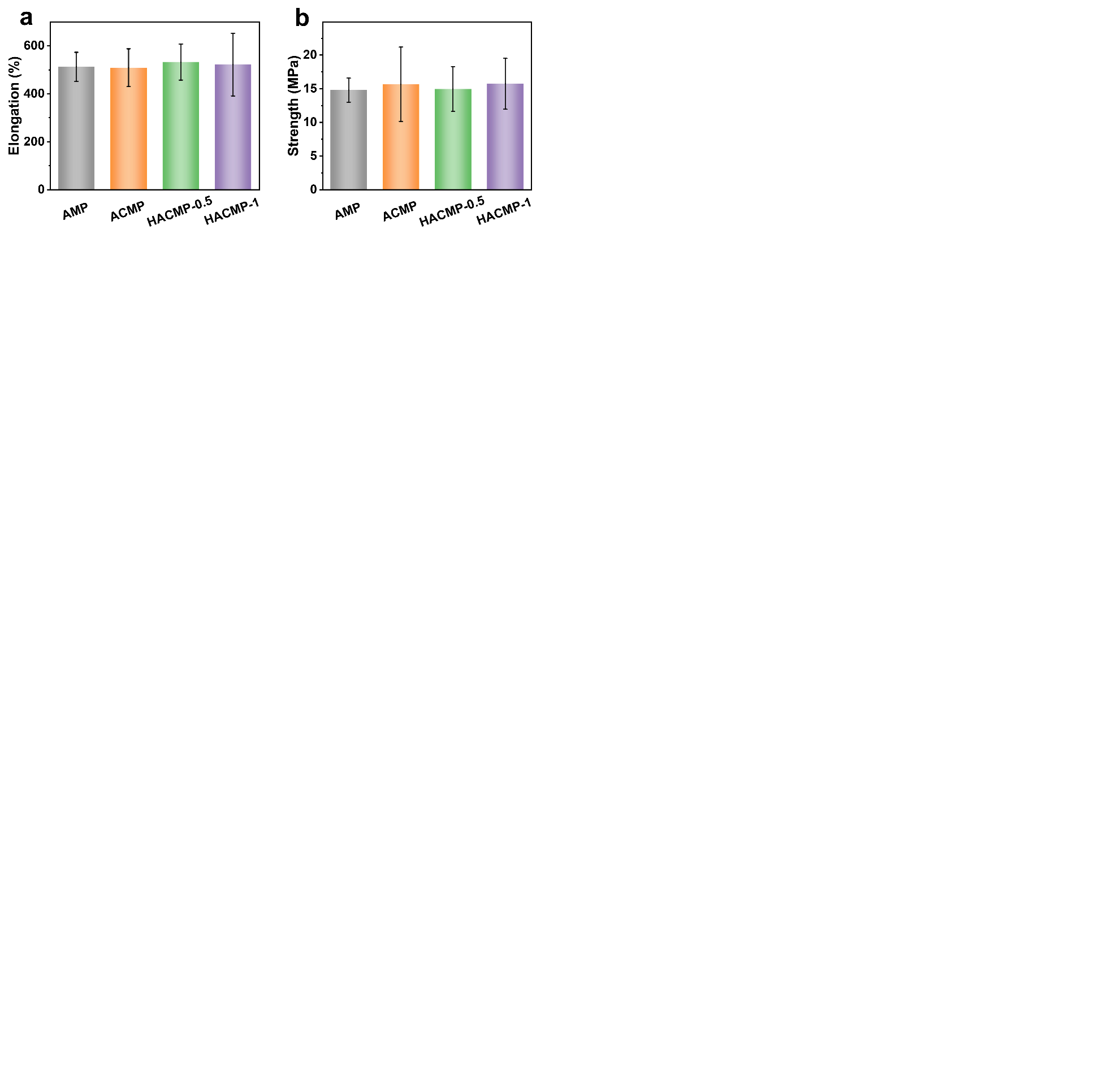
**

**Figure S5** (a) Elongation at break and (b) strength of the patches of patches.

The striated structure does not significantly affect the breaking strength and elongation at break of the patches. This is attributed to two key factors: The highly elastic polyurethane (PU) fiber bundles (break elongation >400%) serve as the structural backbone, while the striated polypyrrole (PPy) coating, though stretchable, has a lower elongation at break (~200%). The composite behavior is therefore governed by the PU matrix, with the PPy layer contributing minimally to the overall mechanical properties. In addition, the patches underwent pre-strain of less than 50% strain, which the plastic deformation of the PU fibers was negligible, so the damage to the PU fibers due to the pre-strain engineering could be negligible. This explains the similarity of elongation at break and breaking strength among different patches.


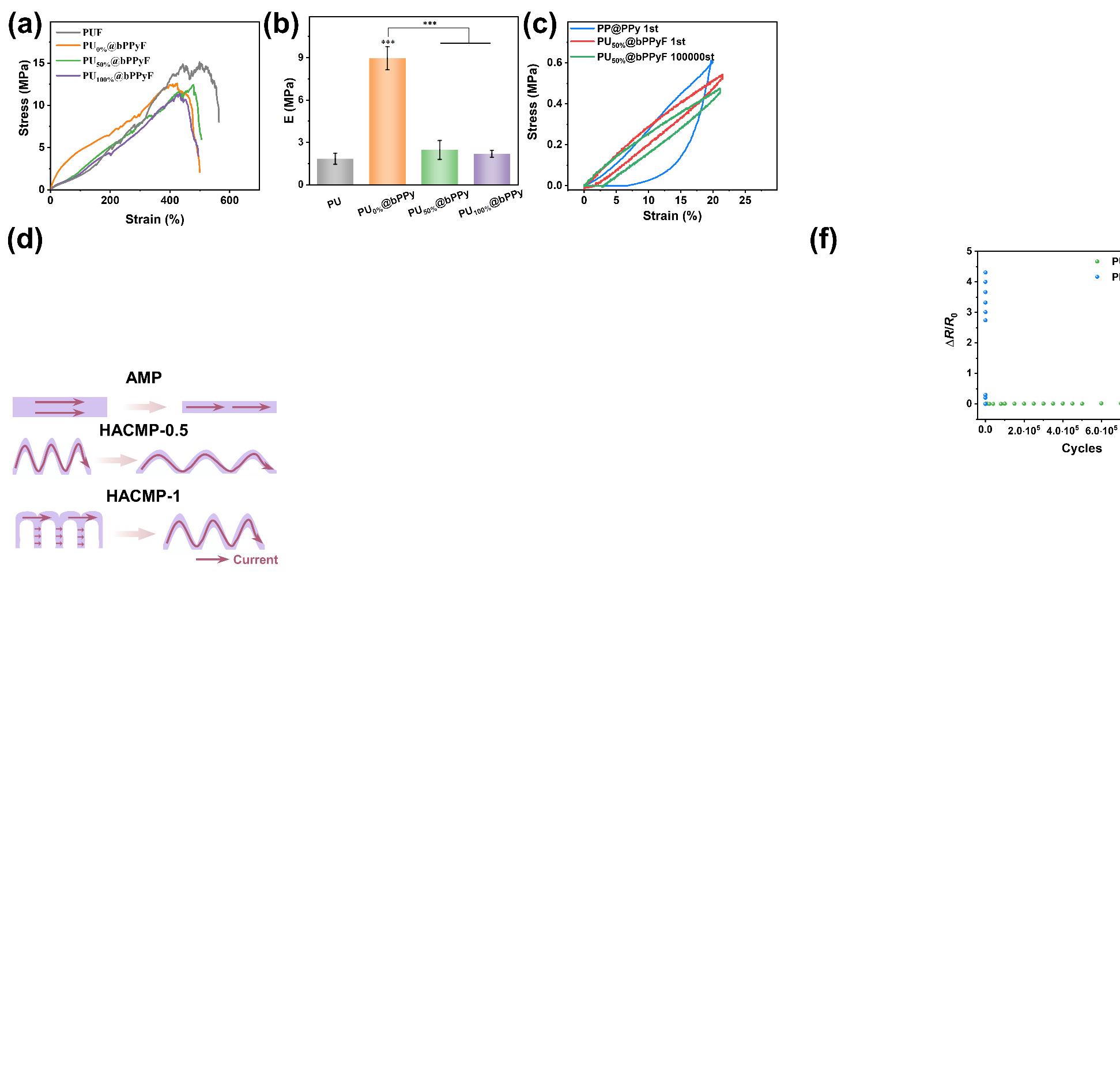


**Figure S6** Structural change of the conductive coating of ACMP and HACMP under stretching strain.

**
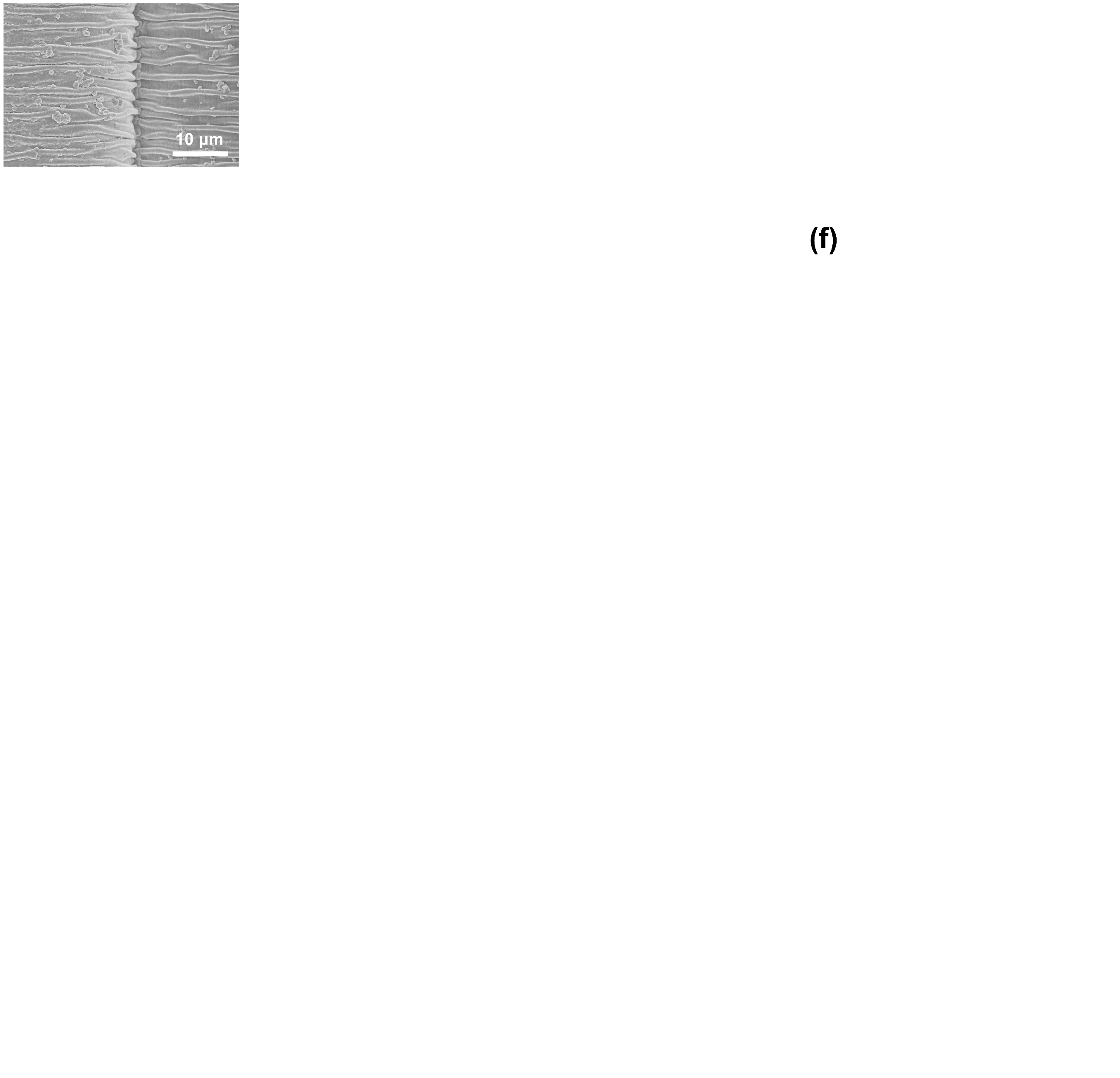
**

**Figure S7** SEM of HACMP-0.5 after 1,000,000 cycles.

**Figure S8** Relative resistance changes under stretching of HACMP-0.5 before and after 1,000,000 cycles.

**
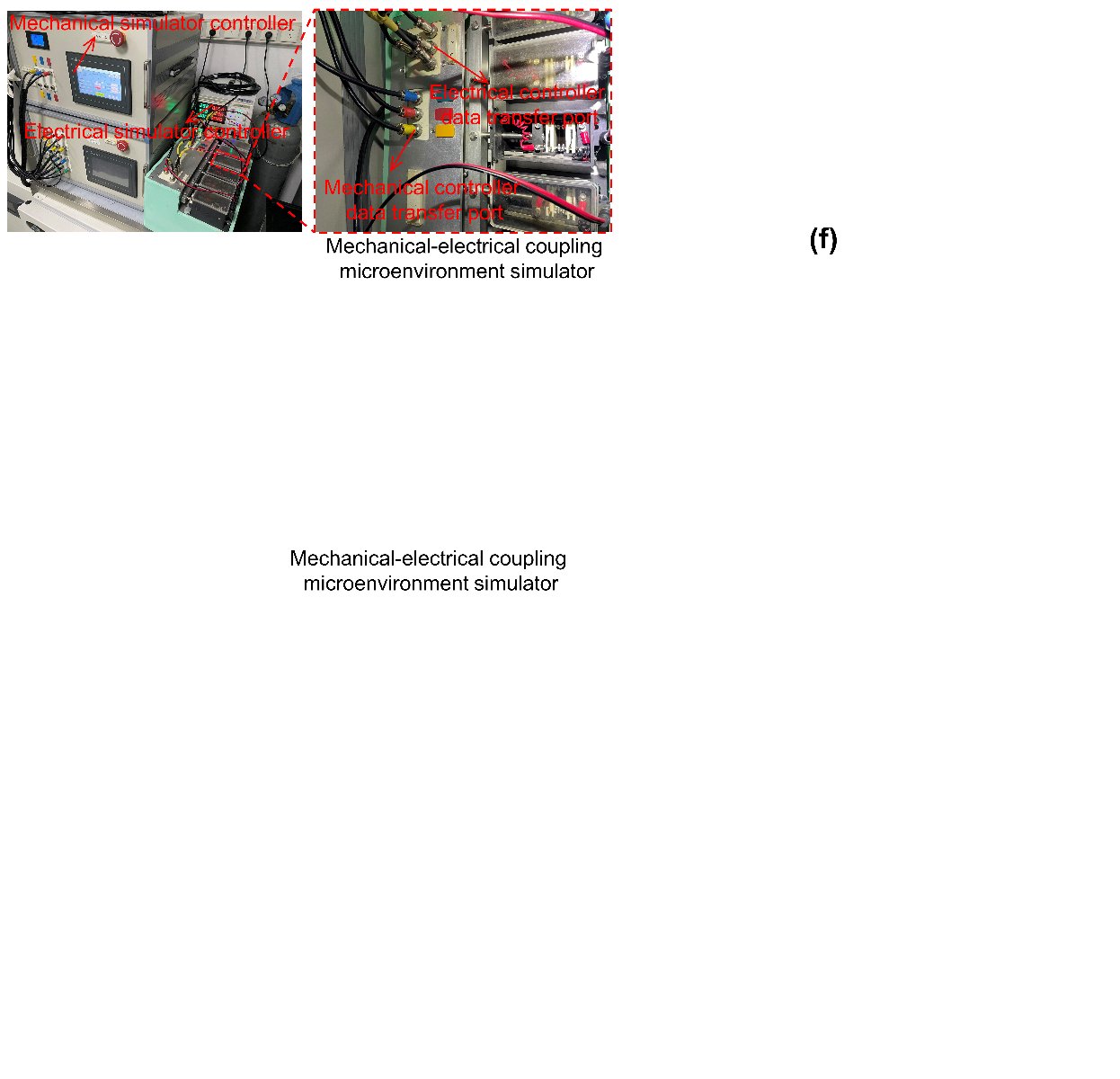
**

**Figure S9** Optical images of a coupled mechanical-electrical microenvironmental simulator.


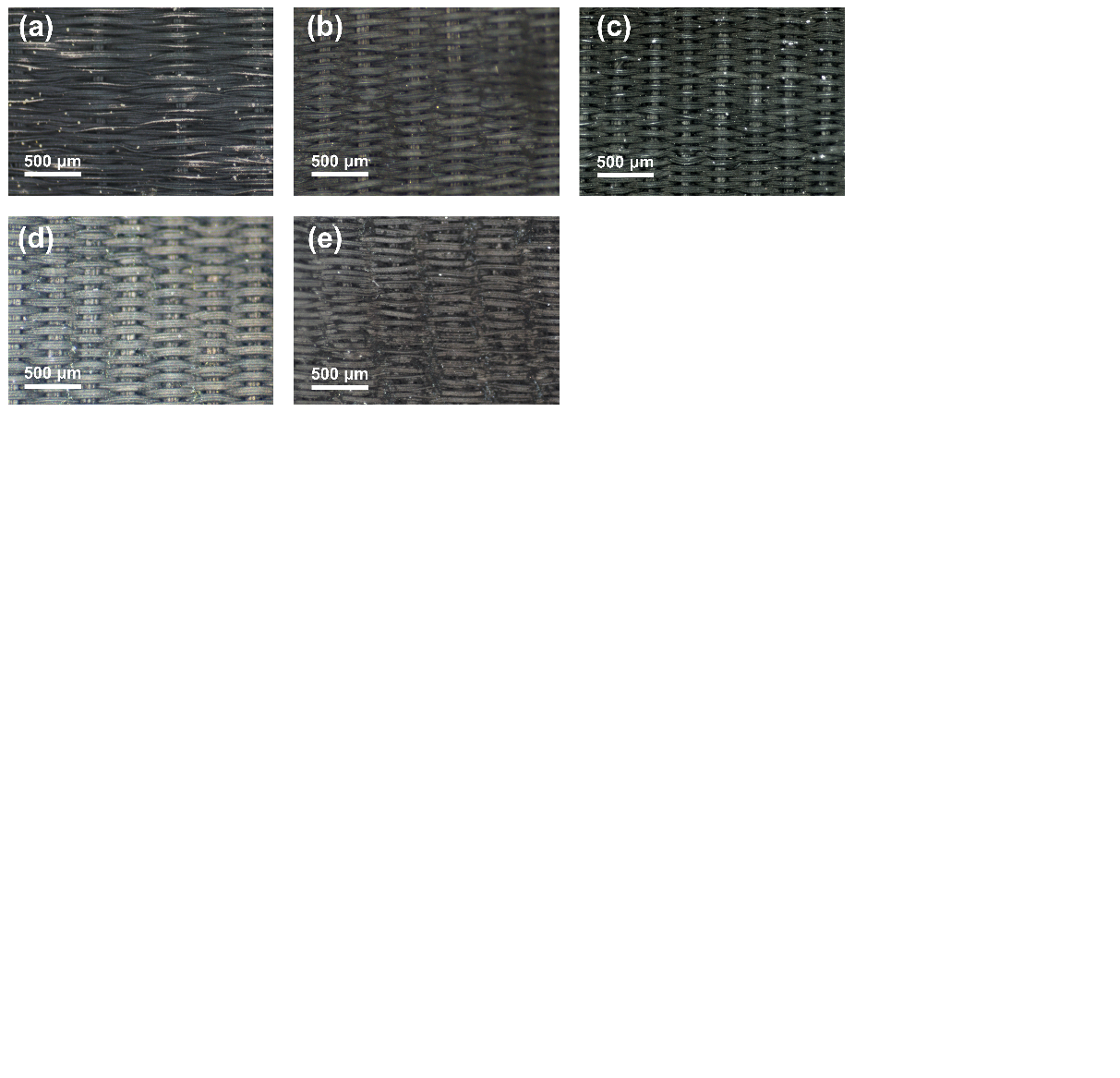


**Figure S10** Optical images of HACMP-0.5 with different weaving parameters: (a) warp 150 cm^-1^, weft 10 cm^-1^; (b) warp 150 cm^-1^, weft 15 cm^-1^; (c) warp 150 cm^-1^, weft 20 cm^-1^; (d) warp 100 cm^-1^, weft 15 cm^-1^; (e) warp 200 cm^-1^, weft 15 cm^-1^.


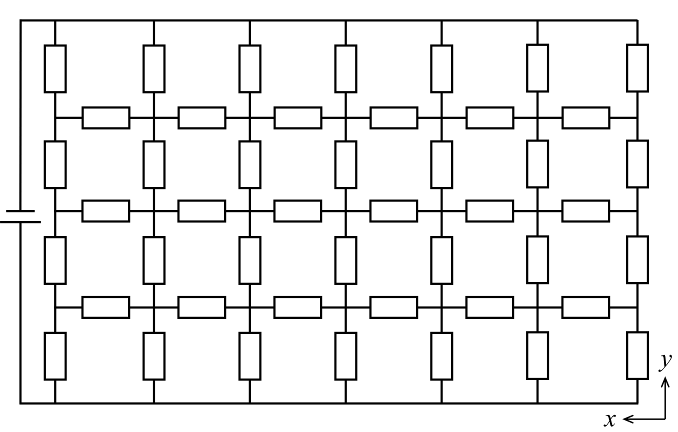


**Figure S11** Resistive equivalent modeling of myocardial patches.


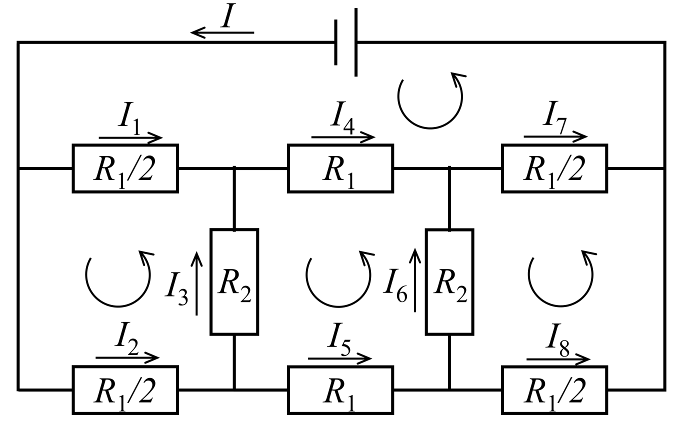


**Figure S12** Resistive equivalent modeling of a fabric structural unit of a myocardial patch.


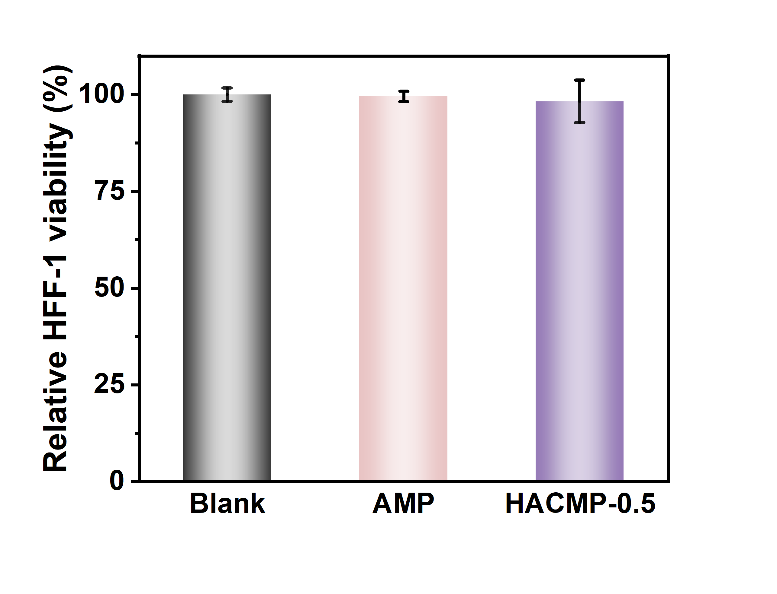


**Figure S13** Cytotoxicity of cardiac patches.

**Supplementary Methods**

The myocardial patches based on the weaving technology undergo several main stages when subjected to tensile stress: Under the action of tensile stress, firstly the bended yarn caused by the warp and weft interlacing gradually becomes straightened. Then the unwound yarns are stretched. It should be noted that there is friction between the interlacing points of warp and weft yarns during this process. The fibers gradually break until the textile-based patches break. In the first stage, the bending of the yarns due to the interlacing structure is related to the fabric density and the fabric structure. Therefore, the main contributing sources to the Young's modulus of the textile-based patches are: the straightening of fiber bending, fiber stretching (mainly manifested as Young's modulus), and the friction between fibers, which can be expressed by Equation S1.

$\left\{\begin{matrix} E_{1}=\frac{\alpha_{1}E_{\text{f1}}s_{1}m_{1}}{d_{F}}+\beta m_{1}m_{2} \\ E_{2}=\frac{\alpha_{2}E_{\text{f2}}s_{2}m_{2}}{d_{F}}+\beta m_{1}m_{2} \end{matrix}\right.$ （S1）

Where, E_1_ and E_2_ are the warp and weft Young's moduli of the myocardial patch respectively, α is a parameter related to the myocardial patch in the first tensile stage, E_f1_ and E_f2_ are parameters characterizing the tensile strength of warp and weft yarns respectively and are related to the Young's modulus of fibers, m_1_ and m_2_ are the warp and weft densities of the myocardial patch respectively, s_1_ and s_2_ are the cross-sectional areas of warp and weft yarns respectively, d_F_ is the thickness of the myocardial patch, and β is a constant related to the friction between warp and weft yarns. Representing αE_f_ as α’, Equation S2 can be combined and simplified as follows:

$\left\{\begin{matrix} E_{F_{1}}=\frac{\alpha_{1}^{'}s_{1}m_{1}}{d_{F}}+\beta m_{1}m_{2} \\ E_{F_{2}}=\frac{\alpha_{2}^{'}s_{2}m_{2}}{d_{F}}+\beta m_{1}m_{2} \end{matrix}\right.$ （S2）

Among these physical quantities, except for α’ and β, the rest are either known or measurable. Based on the data fitting of Figure 3i, it can be obtained that α_1_’ is 13, α_2_’ is 14, and β is 0.016. Therefore, the mechanical prediction model of the HACMP-0.5 can be expressed by Equation S3. Furthermore, the theoretical values of the Young's modulus and the anisotropy ratio of the HACMP-0.5 with the same weft density but different warp densities calculated by Equation S3 are highly consistent with the experimental values (Figure 3m & n), which verifies the validity of the mechanical prediction model. Here, the units of all variables related to dimensions are in millimeters.

$\left\{\begin{matrix} E_{F_{1}}=0.250m_{1}+0.016m_{1}m_{2} \\ E_{F_{2}}=0.137m_{2}+0.016m_{1}m_{2} \end{matrix}\right.$ （S3）

The resistance of the myocardial patch mainly consists of two parts: the length resistance of the yarn and the contact resistance at the interlacing points. In the first part, assuming that the warp and weft yarns are interlaced perfectly perpendicular to each other, when ignoring the contact resistance at the interlacing points and only considering the length resistance of the warp and weft yarns, the resistive equivalent model of the conductive myocardial patch is shown in Figure S6. The resistive equivalent model of a simplified unit of this model is shown in Figure S7.

According to Kirchhoff's laws, the following equation can be obtained:

$\left\{\begin{matrix} R_{F_{1}}I-\frac{1}{2}R_{1}I_{1}-R_{1}I_{4}-\frac{1}{2}R_{1}I_{7}=0 \\ \frac{1}{2}R_{1}I_{2}+R_{2}I_{3}-\frac{1}{2}R_{1}I_{1}=0 \\ R_{2}I_{6}+R_{1}I_{5}-R_{1}I_{4}-R_{2}I_{3}=0 \\ \frac{1}{2}R_{1}I_{8}+R_{2}I_{6}-\frac{1}{2}R_{1}I_{7}=0 \end{matrix}\right.$ （S4）

According to the loop law, Equation S5 can be obtained:

$\left\{\begin{matrix} I=i_{1} \\ I_{1}=i_{1}-i_{2} \\ I_{2}=i_{2} \\ I_{3}=i_{2}-i_{3} \\ I_{4}=i_{1}-i_{3} \\ I_{5}=i_{3} \\ I_{6}=i_{3}-i_{4} \\ I_{7}=i_{1}-i_{4} \\ I_{8}=i_{4} \end{matrix}\right.$ （S5）

By simultaneously solving Equation S4 and Equation S5, it can be concluded that the resistance R_F1_ in the warp direction of the myocardial patch is R_1_. Similarly, the resistance R_F2_ in the weft direction of the myocardial patch is R_2_. The contact resistance part at the interlacing points is related to the densities of the warp and weft yarns and can be simplified as λm_1_m_2_, where λ is related to the contact resistance of the interlacing points of the warp and weft yarns. Therefore, the resistance of the textile-based myocardial patch can be calculated by Equation S6.

$\left\{\begin{matrix} R_{F_{1}}=\frac{R_{1}^{'}}{m_{1}}+\lambda m_{1}m_{2} \\ R_{F_{2}}=\frac{R_{2}^{'}}{m_{2}}+\lambda m_{1}m_{2} \end{matrix}\right.$ （S6）

Where, R’_1_ and R’_2_ are the resistance values per unit length of the warp and weft yarns respectively.

Based on the electrical conductivity calculation Equation S7 and combined with Equation S6, the calculation Equations S8 for the electrical conductivities σ_1_ and σ_2_ in the warp and weft directions of the myocardial patch are obtained.

$\sigma=\frac{l}{Rs_{F}}$ （S7）

Where, σ is the electrical conductivity of the material, *l* is the length of the patch, R is the resistance of the patch, and s_F_ is the cross-sectional area of the patch.

$\left\{\begin{matrix} \sigma_{1}=\frac{m_{1}}{(R_{1}+\lambda m_{1}^{2}m_{2})d_{F}} \\ \sigma_{2}=\frac{m_{2}}{(R_{2}+\lambda m_{1}m_{2}^{2})d_{F}} \end{matrix}\right.$ （S8）

By fitting the data in Figure 3g, the prediction Equations for the electrical conductivities in the warp and weft directions of the myocardial patch are:

$\left\{\begin{matrix} \sigma_{1}=\frac{m_{1}}{20000+0.02m_{1}^{2}m_{2}} \\ \sigma_{2}=\frac{m_{2}}{500000+0.02m_{1}m_{2}^{2}} \end{matrix}\right.$ （S9）

Furthermore, the theoretical values of the electrical conductivities and the anisotropy ratios of the HACMP-0.5 with the same weft density but different warp densities are calculated by Equation S9, and the results are highly consistent with the experimental values (Figure 3k and i), which verifies the validity of the electrical prediction model. Here, the units of all variables related to dimensions are in centimeters.

**Supplementary Tables**

**Table S1** Chemical state contents of nitrogen (N1s) before and after the incubation.

| HACMP-0.5 | =N^+^ | -N^+^ | -NH- | =N |
| --- | --- | --- | --- | --- |
| Before incubation | 11.08% | 9.34% | 78.98% | 3.60% |
| After incubation | 9.16% | 7.37% | 79.46% | 4.00% |

**Table S2** Elemental contents tested by XPS before and after the incubation of HACMP.

| HACMP-0.5 | S | Cl | C | N | O |
| --- | --- | --- | --- | --- | --- |
| Before incubation | 1.3% | 0.62% | 69.77% | 8.26% | 20.05% |
| After incubation | 1.44% | 0.04% | 67.37% | 5.23% | 25.93% |

**Table S3** Mechanical properties of cardiac patches and cardiac tissue engineering scaffolds

| Composition | Preparation method | Young's modulus | Elastic strain | Anisotropy | Ref. |
| --- | --- | --- | --- | --- | --- |
| Decellularized porcine myocardium | Acellular scaffold | 5.2 ± 1.7MPa | 16.9% | Anisotropy | ^[1]^ |
| Decellularized porcine omentum/Au nanoparticles | Acellular scaffold | ~10 - 15MPa | <10% | N/A | ^[2]^ |
| poly (glycerol sebacate)/poly (ε-caprolactone) | 3D printing | 2.27 ± 0.27 MPa | 18.4 ± 2.4% | N/A | ^[3]^ |
| polycaprolactone/ gelatin | Electrospinning | ~15 kP | N/A | N/A | ^[4]^ |
| poly(ester-urethane)urea | Electrospinning | ~0.95 MPa | ~285% | N/A | ^[5]^ |
| PMECLs/ PPy | 3D printing | 20.47 ± 0.88 MPa | 609.51 ± 10.78 % | N/A | ^[6]^ |
| silicone-poly(lactic-co-glycolic acid) | 3D printing | 350-750 kPa | >50% | Anisotropy | ^[7]^ |
| Polycaprolactone/poly(xylitol sebacate)/MWCNTs | Electrospinning | 19.6 ± 2.4 MPa | <50% | N/A | ^[8]^ |
| gelatin methacrylate | Microfluidic focusing/ parallel packing processes | ~ 500 KPa | N/A | Anisotropy | ^[9]^ |
| Carboxymethyl cellulose/Gelatin /Graphene oxide | 3D printing | 410.87 kPa | <50% | N/A | ^[10]^ |
| poly (CL-co-TOSUO)/collagen (PCT/collagen) core/shell nanofibers | Coaxial electrospinning | 26.27 ± 3.14 MPa | >300% | N/A | ^[11]^ |
| poly(1,8-octamethylene-citrate-co-octanol)/ polyethylene glycol lactide acid diacrylate | Laser cutter | 235 ± 50 kPa | <70% | N/A | ^[12]^ |
| Chitosan/lipoic acid/proanthocyanidins | Hydrogel | 45-60 kPa | N/A | N/A | ^[13]^ |
| PEDOT NPs/ poly gallic acid/ gelatin methacrylamide | Hydrogel | 138 kPa | > 90% | N/A | ^[14]^ |
| Poly(ε-caprolactone) | Melt electrowritten | 2.09 ± 0.26 MPa | 5% | Anisotropy; 2.01 | ^[15]^ |
| Poly(ε-caprolactone) | Melt electrowritten | 5 - 11 MPa | 35-40% | Anisotropy | ^[16]^ |
| Polycarbonate Urethane | Electrospinning | 0.5 - 2MPa | N/A | Anisotropy | ^[17]^ |
| Silk/ Reduced graphene oxide | Electrospinning | 5.8 ± 0.4 MPa | <20% | Isotropic | ^[18]^ |
| Poly (glycerol sebacate) | Casting on micro-patterned molds | 6.2 - 11.6 MPa | 50% | Anisotropy | ^[19]^ |
| Poly(glycerolsebacate)-poly(ε-caprolactone)/VEGF | Electrospinning | 8 ± 2MPa | ~20% | N/A | ^[20]^ |
| Polyurethane/gelatin | Electrospinning | 28 - 192MPa | <10% | N/A | ^[21]^ |
| Chitosan/silk | Electrospinning | 200 - 1000MPa | <5% | N/A | ^[22]^ |
| PPy/ tunic cellulose | Hydrogel | ~ 59.0 MPa | <50% | Anisotropy | ^[23]^ |
| Polycaprolactonediol/1,6-hexamethylen diisocyanate/glucocorticoid methylprednisolone | Electrospinning | 3.15 ± 0.72 MPa | 388.8 ± 28.4% | N/A | ^[24]^ |
| Adeno-associated virus /polyester urethane urea /polyester ether urethane urea | Electrospinning | 1.1 ± 0.1 MPa | 292 ± 12% | N/A | ^[25]^ |
| HACMP-0.5 | Weaving | 0.39 - 3.27 MPa | >400% | Anisotropy;  1.8-5.1 tunable | This work |

PMECLs: Poly(γ-methyl-Ɛ-caprolactone)-b-poly (ε-caprolactone)-PEG-poly (ε-caprolactone) -b-poly(γ-methyl-Ɛ-caprolactone); MWCNT: multi-walled carbon nanotubes; CL: ε-caprolactone; TOSUO: 1, 4, 8-trioxaspiro-[4,6]-9-undecanone;

**Table S4** Conductive properties of cardiac patches

| Composition | Preparation method | Conductivity  （S cm^-1^） | Anisotropy | Conductive stability  under cyclic strain | Ref. |
| --- | --- | --- | --- | --- | --- |
| PMECLs/PPy | 3D printing | 2.9 × 10^-2^ ± 2.8 × 10^-3^ | N/A | N/A | ^[6]^ |
| Polycaprolactone/poly(xylitol sebacate)/MWCNTs | Electrospinning | 1.2 ± 0.1 × 10^-2^ | N/A | N/A | ^[8]^ |
| gelatin methacrylate | Microfluidic focusing/ion-photocrosslinking/parallel packing processes | 16.4 ± 1.7 / 7.2 ± 1.1 | Anisotropy | N/A | ^[9]^ |
| Carboxymethyl cellulose/Gelatin /Graphene oxide | 3D printing | 7.0 × 10^−3^ | N/A | N/A | ^[10]^ |
| Chitosan/lipoic acid/proanthocyanidins/Eu^3+^ | Hydrogel | 1.3 × 10^−4^ | N/A | 30 mm min^−1^ for 100 cycles and 50 mm min^−1^ for 1000 cycles with 80% deformation | ^[13]^ |
| PEDOT NPs/ poly gallic acid/ gelatin methacrylamide | Hydrogel | >15 | N/A | 60 % deformation for 50 cycles. | ^[14]^ |
| PPy/poly(ε-caprolactone) | Melt electrowritten | 2.4×10^-2^–13.6×10^-2^ | Anisotropy | Cyclic loading in 5% increments until ε = 20%, *σ*/*σ*_0_=40% | ^[15]^ |
| Silk/ Reduced graphene oxide | Electrospinning | 0.2-0.3 | Anisotropy; 1.7 | N/A | ^[18]^ |
| MXene Ti_2_C/GelMA | Hydrogel | 4-8×10^-4^ | N/A | 60 % deformation for 100 cycles. | ^[26]^ |
| Chitosan/tannic acid/Cu^2+^ | One-step method | 10.0 × 10^−1^ | Anisotropy | N/A | ^[27]^ |
| Acrylamide/acrylic acid/ glycidyl methacrylate | Hydrogel | 0.2-0.45 × 10^−2^ | N/A | 120 % deformation for 100cycles | ^[28]^ |
| Polyacrylic acid/oxidized alginate/gelatin | Hydrogel | 3.5×10^-2^ | N/A | 52.12% deformation for 100 cyclic compressions | ^[29]^ |
| HACMP-0.5 | Weaving | 3×10^-5^–4×10^-4^ | Anisotropy;  2.1-8.3 tunable | Δ*R*/*R*_0_ of fiber exhibited only a 0.01 increase after 1 million cycles | This work |

**Supplementary References**

[1] Wang B, Borazjani A, Tahai M, Curry ALdJ, Simionescu DT, Guan J, et al. Fabrication of Cardiac Patch with Decellularized Porcine Myocardial Scaffold and Bone Marrow Mononuclear Cells. Journal of Biomedical Materials Research Part A. 2010;94A(4):1100-10.

[2] Shevach M, Fleischer S, Shapira A, Dvir T. Gold Nanoparticle-Decellularized Matrix Hybrids for Cardiac Tissue Engineering. Nano Letters. 2014;14(10):5792-6.

[3] Qian B, Shen A, Huang S, Shi H, Long Q, Zhong Y, et al. An Intrinsically Magnetic Epicardial Patch for Rapid Vascular Reconstruction and Drug Delivery. Advanced Science. 2023;10(36):2303033.

[4] Jarrell D, Jacot J. An in Vitro Characterization of a Pcl-Fibrin Scaffold for Myocardial Repair. Materials Today Communications. 2023;37:107596.

[5] Liu Y, Wang L, Liu Z, Kang Y, Chen T, Xu C, et al. Durable Immunomodulatory Nanofiber Niche for the Functional Remodeling of Cardiovascular Tissue. ACS Nano. 2023;18(1):951-71.

[6] Huang W, Xiao Y, Yin W, Yang S, Lang M. An Engineered Cardiac Patch Based on Biodegradable Thermoplastic Elastomer Fabricated by 3d Printing and in Situ Polymerization. European Polymer Journal. 2024;209:112890.

[7] Lou L, Rubfiaro A, Deng V, He J, Thomas T, Roy M, et al. Harnessing 3d Printing and Electrospinning for Multiscale Hybrid Patches Mimicking the Native Myocardium. ACS Applied Materials & Interfaces. 2024;16(29):37596-612.

[8] Sigaroodi F, Boroumand S, Rahmani M, Rabbani S, Hosseinzadeh S, Soleimani M, et al. Mwcnt-Loaded Pcl/Pxs-Pcl Bilayer Cardiac Patch for Myocardial Regeneration: An in Vitro and in Vivo Study. Journal of Polymers and the Environment. 2024;32(11):5994-6010.

[9] Jia X, Liu W, Ai Y, Cheung S, Hu W, Wang Y, et al. A Multifunctional Anisotropic Patch Manufactured by Microfluidic Manipulation for the Repair of Infarcted Myocardium. Advanced Materials. 2024;36(44):2404071.

[10] Arici S, Kamali A, Ege D. Cmc/Gel/Go 3d-Printed Cardiac Patches: Go and Cmc Improve Flexibility and Promote H9c2 Cell Proliferation, While Edc/Nhs Enhances Stability. Biofabrication. 2025;17(1):015025.

[11] Wang Y, Fan Z, Li Q, Lu J, Wang X, Zhang J, et al. Construction of a Myocardial Patch with Mesenchymal Stem Cells and Poly(Cl-Co-Tosuo)/Collagen Scaffolds for Myocardial Infarction Repair by Coaxial Electrospinning. Journal of Materials Chemistry B. 2023;11(22):4980-90.

[12] Ryu H, Wang X, Xie Z, Kim J, Liu Y, Bai W, et al. Materials and Design Approaches for a Fully Bioresorbable, Electrically Conductive and Mechanically Compliant Cardiac Patch Technology. Advanced Science. 2023;10(27):2303429.

[13] Li Z, Li Q, Cao W, Zhan J, He Y, Xing X, et al. A Strongly Robust Chitosan-Based Programmed Control Functional Hydrogel Improved Mitochondrial Function and Pro-Vascularization for Adaptive Repair of Myocardial Infarction. Advanced Functional Materials. 2024;34(16):2312631.

[14] Chen P, Zhang W, Fan X, Shi X, Jiang Y, Yan L, et al. A Polyphenol-Derived Redox-Active and Conductive Nanoparticle-Reinforced Hydrogel with Wet Adhesiveness for Myocardial Infarction Repair by Simultaneously Stimulating Anti-Inflammation and Calcium Homeostasis Pathways. Nano Today. 2024;55:102157.

[15] Olvera D, Molina MS, Hendy G, Monaghan MG. Electroconductive Melt Electrowritten Patches Matching the Mechanical Anisotropy of Human Myocardium. Advanced Functional Materials. 2020;30(44):1909880.

[16] Castilho M, van Mil A, Maher M, Metz CHG, Hochleitner G, Groll J, et al. Melt Electrowriting Allows Tailored Microstructural and Mechanical Design of Scaffolds to Advance Functional Human Myocardial Tissue Formation. Advanced Functional Materials. 2018;28(40):1803151.

[17] Ayaz HGS, Perets A, Ayaz H, Gilroy KD, Govindaraj M, Brookstein D, et al. Textile-Templated Electrospun Anisotropic Scaffolds for Regenerative Cardiac Tissue Engineering. Biomaterials. 2014;35(30):8540-52.

[18] Zhao G, Feng Y, Xue L, Cui M, Zhang Q, Xu F, et al. Anisotropic Conductive Reduced Graphene Oxide/Silk Matrices Promote Post-Infarction Myocardial Function by Restoring Electrical Integrity. Acta Biomaterialia. 2022;139:190-203.

[19] Shi M, Bai L, Xu M, Li Z, Hu T, Hu J, et al. Micropatterned Conductive Elastomer Patch Based on Poly (Glycerol Sebacate)-Graphene for Cardiac Tissue Repair. Biofabrication. 2022;14(3):035001.

[20] Rai R, Tallawi M, Frati C, Falco A, Gervasi A, Quaini F, et al. Bioactive Electrospun Fibers of Poly(Glycerol Sebacate) and Poly(E-Caprolactone) for Cardiac Patch Application. Advanced Healthcare Materials. 2015;4(13):2012-25.

[21] Xie J, Yao Y, Wang S, Fan L, Ding J, Gao Y, et al. Alleviating Oxidative Injury of Myocardial Infarction by a Fibrous Polyurethane Patch with Condensed Ros-Scavenging Backbone Units. Advanced Healthcare Materials. 2022;11(4):2101855.

[22] Chen J, Zhan Y, Wang Y, Han D, Tao B, Luo Z, et al. Chitosan/Silk Fibroin Modified Nanofibrous Patches with Mesenchymal Stem Cells Prevent Heart Remodeling Post-Myocardial Infarction in Rats. Acta Biomaterialia. 2018;80:154-68.

[23] He Y, Hou H, Wang S, Lin R, Wang L, Yu L, et al. From Waste of Marine Culture to Natural Patch in Cardiac Tissue Engineering. Bioactive Materials. 2021;6(7):2000-10.

[24] Yao Y, Ding J, Wang Z, Zhang H, Xie J, Wang Y, et al. Ros-Responsive Polyurethane Fibrous Patches Loaded with Methylprednisolone (Mp) for Restoring Structures and Functions of Infarcted Myocardium <I>in Vivo</I>. Biomaterials. 2020;232.

[25] Gu X, Matsumura Y, Tang Y, Roy S, Hoff R, Wang B, et al. Sustained Viral Gene Delivery from a Micro-Fibrous, Elastomeric Cardiac Patch to the Ischemic Rat Heart. Biomaterials. 2017;133:132-43.

[26] Ye G, Wen Z, Wen F, Song X, Wang L, Li C, et al. Mussel-Inspired Conductive Ti2c-Cryogel Promotes Functional Maturation of Cardiomyocytes and Enhances Repair of Myocardial Infarction. Theranostics. 2020;10(5):2047-66.

[27] Tang G, Li Z, Ding C, Zhao J, Xing X, Sun Y, et al. A Cigarette Filter-Derived Biomimetic Cardiac Niche for Myocardial Infarction Repair. Bioactive Materials. 2024;35:362-81.

[28] Shi T, Wang P, Ren Y, Zhang W, Ma J, Li S, et al. Conductive Hydrogel Patches with High Elasticity and Fatigue Resistance for Cardiac Microenvironment Remodeling. ACS Applied Materials & Interfaces. 2023;15(11):14005-18.

[29] Song X, Wang X, Zhang J, Shen S, Yin W, Ye G, et al. A Tunable Self-Healing Ionic Hydrogel with Microscopic Homogeneous Conductivity as a Cardiac Patch for Myocardial Infarction Repair. Biomaterials. 2021;273:120811.
